# In vivo genome-wide CRISPR screening identifies ZNF24 as a negative NF-κB modulator in lung cancer

**DOI:** 10.1186/s13578-022-00933-0

**Published:** 2022-12-01

**Authors:** Lu Liu, Yuxi Lei, Wensheng Chen, Qian Zhou, Zongyao Zheng, Guandi Zeng, Wanting Liu, Pengju Feng, Zhiyi Zhang, Lei Yu, Liang Chen

**Affiliations:** 1 MOE Key Laboratory of Tumor Molecular Biology and Key Laboratory of Functional Protein Research of Guangdong Higher Education Institutes, Institute of Life and Health Engineering, College of Life Science and Technology, Jinan University, Guangzhou, 510632 China; 2 Department of Chemistry, Jinan University, Guangzhou, 510632 China; 3 Beijing Tongren Hospital, Capital Medical University, Beijing, 100730 China

**Keywords:** Lung cancer, Tumor suppressor gene, ZNF24, NF-κB, Combination therapy

## Abstract

Systemic identification of tumor suppressor genes (TSGs) and elucidation of their signaling provide a new angle for understanding of tumorigenesis, which is important for developing successful treatment for lung cancer patients. In our current work, we conducted an in vivo screen for lung cancer TSGs through CRISPR/Cas9 mediated knockout of genes at genome-wide scale. We found that *ZNF24* was a potent and clinically relevant TSG of lung cancer. Ectopic expression of ZNF24 arrested lung cancer cells in S phase. Mechanistically, ZNF24 bound to promoter region of *P65* to negatively regulate its transcription and thereby the signaling activity of NF-κB pathway. This signaling cascade is clinically relevant. Importantly, we found that combinational inhibition of KRAS, NF-κB, and PD-1 effectively shrank autochthonous *Kras*^G12D^/*ZNF24*^−/−^ lung cancers in transgenic mouse model. Our current work thus revealed an important role played by loss of function of *ZNF24* in lung tumorigenesis and shed new light in precision medicine for a portion of lung cancer patients.

**Supplementary Information:**

The online version contains supplementary material available at 10.1186/s13578-022-00933-0.

## Introduction

Lung cancer, including small cell lung cancer (SCLC) and non‐small cell lung cancer (NSCLC), ranks top among the most frequently diagnosed cancer types. It’s the leading cause of cancer-related death, claiming estimated 1.8 million lives worldwide as of year 2020 [1]. Due to the asymptomatic nature during early stages, approximately 75% of NSCLC patients are diagnosed at an advanced stage [2]. Despite of progress of multimodality therapies in clinic for chemotherapy [3], targeted therapy [4] and immunotherapy [5], prognosis of lung cancer patients remains dismal with a overall 5-year survival of around 20% [1]. The tumorigenesis and development of lung cancer are the ultimate outcome of complex interaction between genetic and environmental factors [6]. Elucidation of critical molecular events during tumorigenesis and delineation of mechanisms underlying the malignant nature of lung cancer cells are expected to shed new light on novel therapies for lung cancers.

Zinc finger protein 24 (*ZNF24*; also known as *KOX17*, *Zfp191* or *ZNF191*), a member of the Kruppel-like zinc finger transcription factor family, features 4 C-terminal C2H2 zinc finger motifs for DNA binding and an N-terminal SCAN domain [7]. The SCAN domain was reported to be involved in protein–protein, protein-DNA and protein-RNA interactions [7]. Members of ZNF family bind to the TCAT repeat, sequence variation of which has quantitative silencing effects on gene expression in vitro and correlates with quantitative and qualitative changes in ZNF protein binding [8]. *ZNF24* mRNA is expressed in various organs during embryonic development and adulthood. Several negative transcriptional targets have been reported for ZNF24, including *VEGF* and *PDGFRB* [9–11]. Literature has shown that ZNF24 impacts the proliferation of neural progenitor cells or maintains them in an undifferentiated state [12, 13].

The NF-κB signaling pathway has been reported to be critically involved in various physiological processes, including inflammatory responses, proliferation, differentiation, cell adhesion and apoptosis [14]. Classical and non-classical signaling pathways have been reported for NF-κB signaling. In the classical pathway, IκB kinase (IKK) phosphorylates IκBα at two N-terminal serine sites, which trigger its ubiquitination and proteasome degradation; this leads to release and the ensuing nuclear translocation of NF-κB complexes, predominantly P65/RelA and P50/c-Rel dimers. NF-κB complexes thus bind to the promoters of the target genes involved in a variety of pathways, including *CCND1* and *PTEN* for cell cycle, *VEGF*, *IL-8*, *Ang-2* for angiogenesis, and *ICAM-1*, *MMP9* for invasion and metastasis [15–17]. The NF-κB pathway is regulated by large amounts of signals at many levels [18], abnormalities of which are implicated in tumorigenesis and cancer development [19]. Interestingly, activation of NF-κB has been reported to actively shape tumor microenvironment of lung cancer through upregulating the expression of inflammatory cytokines like IL-6 and activation of STAT3 signaling [20]. Naturally, activation of NF-κB has been reported to play an important role in tumorigenesis and development of lung cancer [16, 21].

Despite of their important role during lung cancer development and in mediating cancer cells’ response to therapeutics, TSGs in lung cancer remain to be systemically determined. Here, we conducted a genome-wide screening for identifying TSGs through CRISPR/Cas9 mediated knockout. We report that *ZNF24* is a potential tumor suppressor gene. Deficiency of *ZNF24* resulted in activation of NF-κB signaling pathway in lung cancer cells. *ZNF24* exerted potent tumor suppressive roles both in lung cell lines and in mouse models of xenograft and autochthonous lung cancers. Mechanistically, ZNF24 negatively regulated the transcription of *P65* by binding to its promoter. Importantly, we found that NF-κB inhibitor, pan-KRAS inhibitor and anti-PD-1 synergized to treat lung cancer deficient of *ZNF24*.

## Results

### ZNF24 is an essential tumor suppressor in lung cancer

**Fig. 1:**
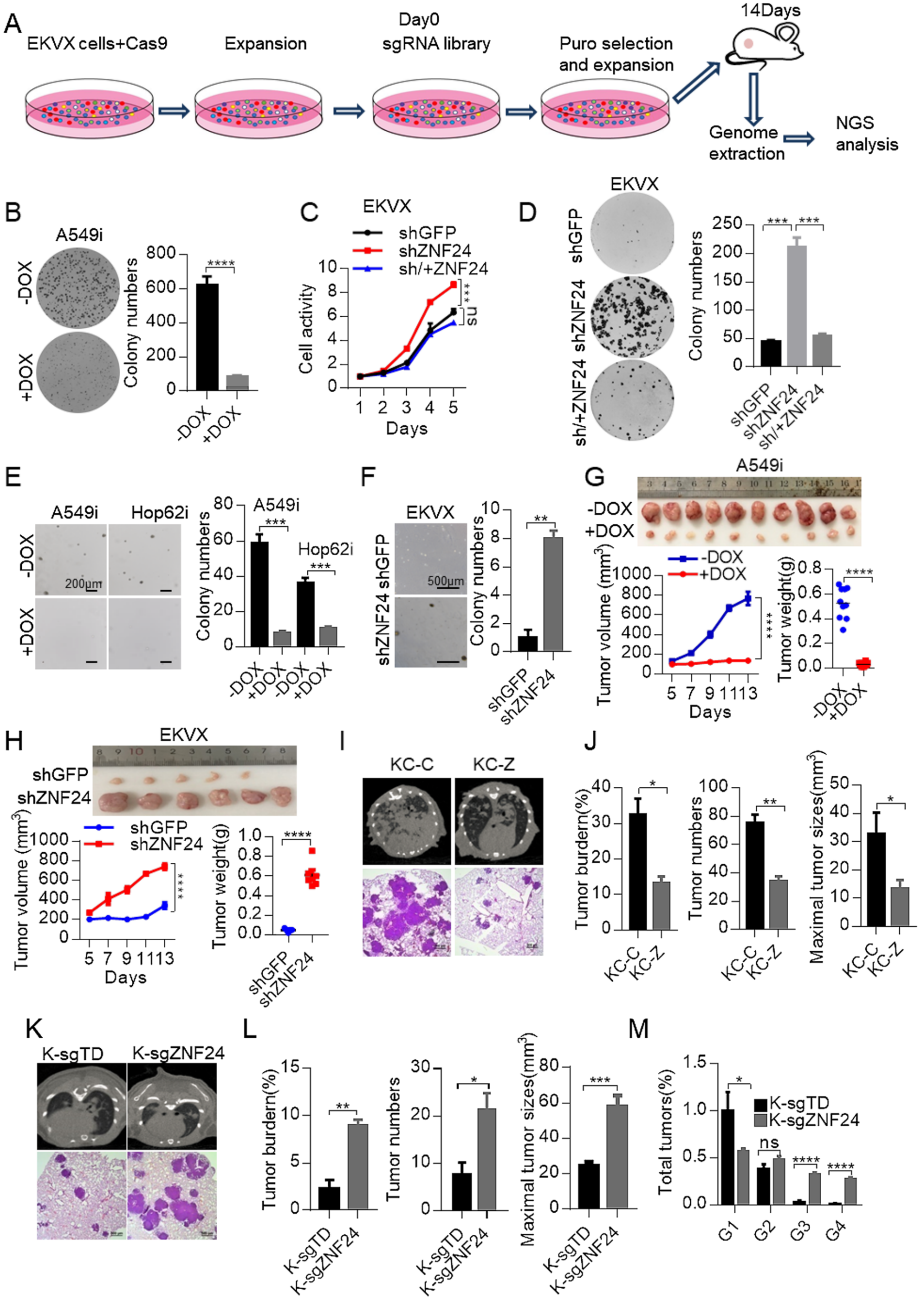
*ZNF24* is an essential tumor suppressor in lung cancer. **A** Schematic of CRISPR-based screen for lung cancer suppressor genes. EKVX cells were infected with virus encoding Cas9, and selected with blasticidin for two weeks. Monoclone was picked to generate a stable cell line (EKVX-Cas9). EKVX-Cas9 were infected the sgRNA library with a multiplicity of infection of 0.3. Cells were selected with puromycin for 7 days and then subcutaneously transplanted into nude mice to grow tumor for two weeks. Genomic DNA were isolated from tumors and used as templates to amplify DNA fragment containing CRISPR sequence. PCR products were sequenced. **B** Impact of ZNF24 expression level on colony forming ability of A549i cells. A549i (1000 cells) were seeded in 6-well plates and treated with/without Dox for 2 weeks before quantification of colonies. Left: representative pictures. Right: statistics of colony number. **C** Effect of ZNF24 knockdown or re-expression on proliferation of EKVX cells. 1000 EKVX-shZNF24, sh/ + ZNF24 or shGFP cells were seeded in 96-well plates and cultured for 5 days. Cell viability was analyzed with CCK8. shGFP for control knockdown; shZNF24 for ZNF24 knockdown; sh/ + ZNF24 for ZNF24 re-expression in ZNF24 knockdown cells. **D** Impact of ZNF24 knockdown or re-expression on 2-D colony forming ability of EKVX cells. 1000 engineered EKVX cells were respectively inoculated in 6-well plates and cultured for 2 weeks before quantification for colonies. Left: representative pictures. Right: statistics of colony number. **E** Impact of ZNF24 expression level on soft-agar colony-forming ability of A549 and Hop62 cells. A549i, Hop62i (10,000 cells) cultured in top agar in the presence or absence of Dox (1 μg/mL) for 3–4 weeks before quantification for colonies. Scale Bar = 200 μm. left: representative images of colonies. right: statistics. **F** Impact of ZNF24 knockdown on soft-agar colony-forming ability of EKVX cells. 10,000 engineered EKVX cells were respectively inoculated in soft agar in 6 well-plates and cultured 3–4 weeks before quantification for colonies. Scale Bar = 500 μm. left: representative images of colonies. right: statistics. **G** Impact of ZNF24 expression on A549i xenografted tumors. A549i cells (2 × 10^6^) were inoculated into nude mice. Mice treated with Dox (n = 4) or control diet (n = 4) for 14 days. A representative image of the tumors at the end of the experiments is shown. Tumors were measured with a vernier caliper every 2 days. Tumor volume (mm^3^) is calculated by D × d^2^/2, where D is the longest diameter and d is the shortest diameter. Growth curve of the tumor is shown. **H** Impact of Knockdown the ZNF24 on EKVX xenografted tumors. The EKVX-shGFP and EKVX-shZNF24 (2 × 10^6^ cells) were inoculated into nude mice for tumor growth for 14 days. Tumor volume is recorded every 2 days. A representative image of the tumors at the end of the experiments is shown. **I** Impact of ZNF24 expression on autochthonous lung tumor formation in transgenic mice. TetO-Kras^G12D^/CC10rtTA (designated KC) mice infected with virus for expressing control (KC-C) or ZNF24 (KC-Z) and were fed with Dox diet for 2 months. Tumor burdens were recorded through computed tomography (CT) scanning and histological sections. Scale Bar = 500 μm. **J** Statistics of tumor burden, tumor number, maximal tumor sizes (diameters) were measured. **K** Impact of ZNF24 expression on tumor formation in Lsl-Kras^G12D^ mice. The recombinant lenti-virus co-expressed Cre and CRISPR/Cas9 to infect Lsl-Kras^G12D^ by nasal inhalation. Comparison of lung tumor formation in K-sgZNF24 mice and K-sgTD mice six months after infection. Tumor burdens were recorded through Computed tomography (CT) scanning and histological sections. Scale Bar = 500 μm. **L**–**M** Statistics of tumor burdens, tumor numbers, maximal tumor sizes (diameters), Grade 1 (G1)- Grade 4 (G4) tumor of **K** were determined by tumor size and heterogeneity of nuclei of tumor cells. Bars are represented as means ± SEM of the indicated number (n) of repeats. *P < 0.05, **P < 0.01, and ***P < 0.001 by Student’s t-test

To explore potential tumor suppressor genes in lung cancer, we performed a genome-wide screening through CRISPR/Cas9 mediated knockout (Fig. 1A). We first generate a lung cancer cell line, EKVX, for stable expression of Cas9 (Additional file 4: Figure S1A) and then infected it with lentivirus-based CRISPR library for genome-scale knockout of human genes (GeCKO v2 library). These infected lung cancer cells (day 0) were allowed to grow xenografted tumor nodules in nude mice for 2 weeks (day 14). We then determined TSGs by checking CRISPRs enriched in xenografted tumor nodules in comparison to those in day-0 cell samples, based on the hypothesis that cells with TSGs knockout grew faster.

This genome-wide screening generated a list of putative TSG candidates (Additional file 4: Figure S1B, Additional file 1: Table S1). Kyoto Encyclopedia of Genes and Genomes (KEGG) analysis of the most 230 enriched genes revealed pathways, including Prolactin signaling pathway, EGFR tyrosine kinase inhibitor resistance, Neurotrophin signaling pathway and Biosynthesis of amino acids (Additional file 4: Figure S1C). Interestingly, typical TSGs, including *NR3C2* [22, 23], *CDH11* [24] and *KLF6* [25] stood out as TSG candidates, indicating validity of our screening. We picked the top 5 genes (*ZNF24*, *NR3C2*, *CST4*, *ARHGDIG* and *CRYBB3*) to confirm their lung cancer suppressive function. MTT assay revealed that knock-down of these 5 genes significantly promoted cell growth (Additional file 4: Figure S1D–I), strongly arguing that our screening enriched TSGs.

Interestingly, we found that *ZNF24* was the top hit in our screening (Additional file 4: Figure S1B). Tumor suppressive function of *ZNF24* has been noticed in breast cancer and gastric cancer [9, 26]. However, its role in lung cancer remains largely to be determined. We, therefore, focused our further experimental efforts on *ZNF24*.

We found that ZNF24 expression in tumor samples and most of the lung cancer cell lines was consistently lower than in para-tumoral lung tissues (Additional file 4: Figure S1J), strongly arguing that *ZNF24* is a clinically relevant TSG for lung cancer.

In order to validate its TSG function, we established A549 and Hop62 stable cell line for doxycycline (Dox)- inducible expression of ZNF24 (referred to as A549i and Hop62i, Additional file 4: Figure S2A). We found that high level expression of ZNF24 significantly inhibited cell growth and decreased plate colony formation of A549i and Hop62i cells (Additional file 4: Figure S2B, C and Fig. 1B). Conversely, ZNF24 knockdown dramatically enhanced cell growth and colony formation of EKVX and H322 cells in 2-D plates (Fig. 1C, D and Additional file 4: Figure S2D–G). Moreover, re-expression of ZNF24 in shZNF24 cells (designated sh/ + ZNF24) reduced the growth rate and plate colony formation to the degree comparable to that of shGFP infected EKVX and H322 cells (Fig. 1C, D and Additional file 4: Figure S2D–G). Similarly, Ectopic expression of ZNF24 led to a significant decrease the colony formation of A549i and Hop62i and knockdown enhanced the colony formation of EKVX cells in soft agar (Fig. 1E, F). These data clearly supported the tumor suppressive function of *ZNF24 *in vitro.

More importantly, xenografted tumors derived from A549i grow significantly more slowly in Dox treated mice than in control diet treated counterpart (Fig. 1G and Additional file 4: Figure S2H). To make a fair comparison of impact of expression level of ZNF24 on the growth rate of xenograft tumors, we overexpressed, control-manipulated and knockdown ZNF24 in EKVX (Additional file 4: Figure S2I, right). Interestingly, expression level reversely correlated the growth rate of xenograft tumor (Fig. 1H and Additional file 4: Figure S2I–K). Autochthonous lung cancer formation in immune competent mice recapitulate the course of patients’ tumor initiation and development more faithfully than xenograft tumor do in immune deficient mice. Earlier, by crossing TetO-Kras^G12D^ with CC10rtTA, we reported a Dox inducible lung cancer mouse model driven by Kras^G12D^ (designated KC mice) [27]. We then infected these mice with lentivirus containing Teton- ZNF24 elements through intranasal instillation (designated KC-Z). In parallel, we infected KC mice with lentivirus packaged with vacant Teton-Puro vector as control cohort (designated KC-C). IHC and Western blotting analysis confirmed the ectopic ZNF24 expression in lung tissues of Dox treated mice (Additional file 4: Figure S2L). We found that overexpression of ZNF24 significantly inhibited lung cancer formation (Fig. 1I, J). Examination of hematoxylin and eosin (H&E)-stained lung tissue sections revealed retroviral expression of ZNF24 significantly inhibited tumor formation in lungs of KC-Z (Fig. 1I). Conversely, following procedure reported earlier to knockout of TSGs in lung cancer mouse model of Lsl-Kras^G12D^ transgenic mice through CRISPR/Cas9 [28, 29], we intranasally delivered lentivirus targeting either Td-Tomato (serving as a control, K-sgTD) or ZNF24 (K-sgZNF24) into Lsl- Kras^G12D^ mice (Additional file 4: Figure S2M, N). In comparison to control group, we detected significantly more tumor nodules, tumor burdens, larger tumor nodules and tumor size and amount of stage 4 tumors in *ZNF24* knockout mice after 6 months of nasal inhalation of lentivirus (Fig. 1K–M). Taken together, these results convincingly argued that *ZNF24* was a potent novel TSG in vivo.

### Ectopic expression of ZNF24 induces cell-cycle arrest of lung cancer cell

**Fig. 2:**
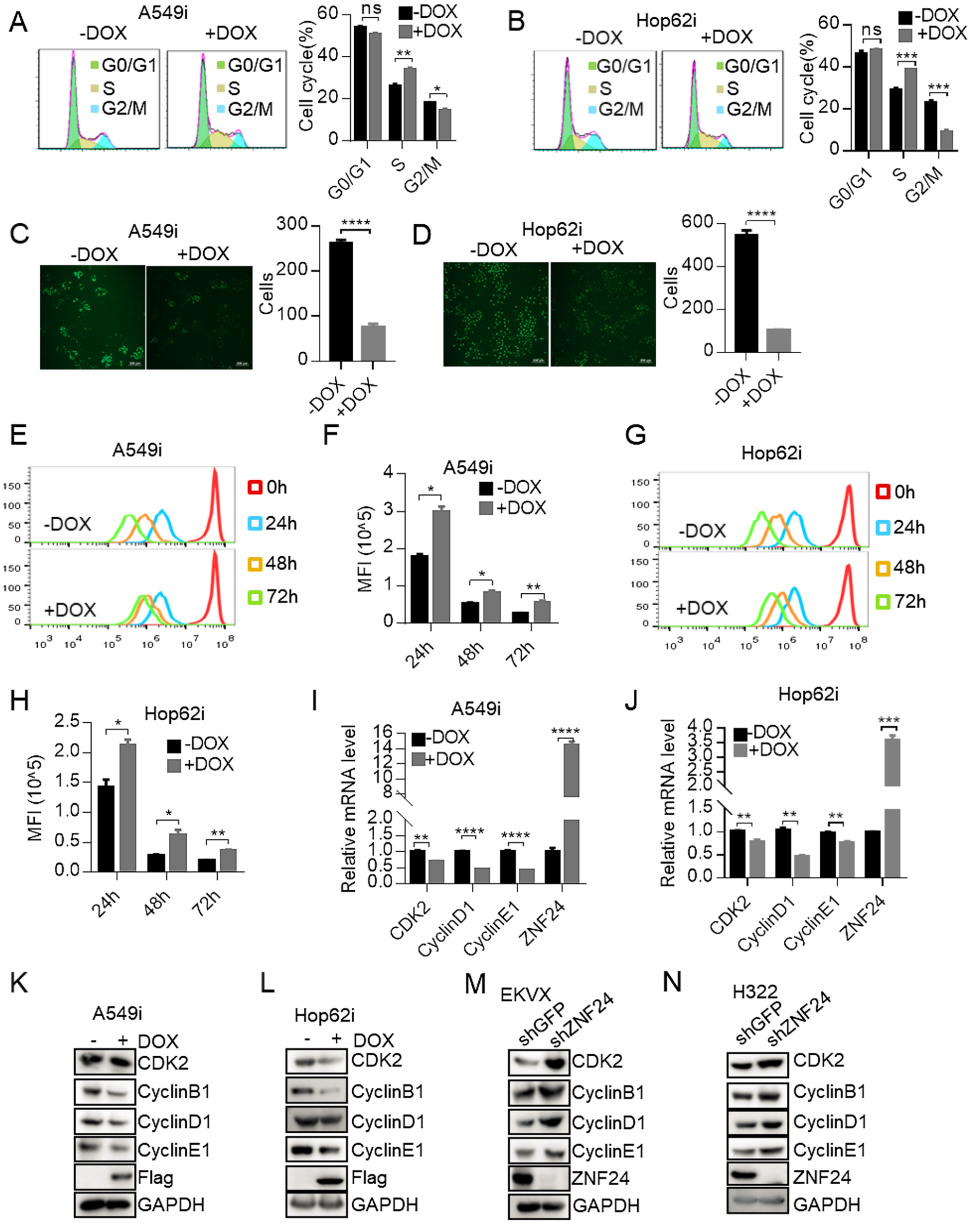
Ectopic expression of ZNF24 induces cell-cycle arrest of lung cancer cell. **A** Impact of ZNF24 expression on cell cycle in A549i cells. A549i cells were treated with Dox (1 μg/mL) for 48 h. Cell cycle were determined through FACS analysis of DNA contents revealed by propidium Iodide (PI) staining. Results are represented as percent of cell population in G0/G1, S and G2/M phases of the cell cycle. **B** Impact of ZNF24 expression on cell cycle of Hop62i cells. Hop62i cells were treated with Dox (1 μg/mL) for 48 h. Propidium iodide (PI)-stained cells were analyzed by FACS. **C** Impact of ZNF24 expression on proliferation of A549i cells. A549i cells were cultured in media containing 1 μg /mL of Dox for 48 h. Edu dye was administered at 10 μM. DNA replication was detected by Edu incorporation through fluorescent microscopy. Scale Bar = 200 μm. **D** Impact of ZNF24 expression on division proliferation of Hop62i cells. Hop62i cells were cultured in media containing 1 μg/mL of Dox for 48 h. Edu dye was administered at 10 μM. DNA replication was detected by Edu incorporation through fluorescent microscopy. Scale Bar = 200 μm. **E** Effect of ZNF24 expression on division proliferation of A549i cells. Cells were stained with 5 μM of CFSE Dye, followed by culture in media containing 1 μg/mL of Dox for 48 h. Cell proliferation as reflected by serial dilution of fluorescence intensity was analyzed by flow cytometry. **F** Statistics of (**E**). **G** Effect of ZNF24 expression on division proliferation of Hop62i cells. Cells were stained with 5 μM of CFSE Dye, followed by culture in media containing 1 μg /mL of Dox for 48 h. Cell proliferation as reflected by serial dilution of fluorescence intensity was analyzed by flow cytometry. **H** Statistics of (**G**). **I** Impact of ZNF24 expression on cell cycle related genes. The A549i cells were induced with Dox for 48 h and RNA was extracted. Expression of genes encoding *CDK2*, *Cyclin D1* and *Cyclin E1* was quantified through RT-qPCR. **J** Impact of ZNF24 expression on cell cycle related genes. The Hop62i cells were induced with Dox for 48 h for RNA extraction. Expression of genes encoding *CDK2*, *Cyclin D1* and *Cyclin E1* was quantified through RT-qPCR. **K**–**N** Impact of ZNF24 on expression of cell cycle-associated proteins in A549i, Hop62i, EKVX and H322 cells through Western blot analysis

To further explore how *ZNF24* exerts its lung cancer suppressive function, we profiled programmed cell death (including apoptosis, pyroptosis and necroptosis), senescence, cell-cycle and DNA damage of lung cancer cells in response to ectopic expression of ZNF24. We found that apoptosis, pyroptosis, necroptosis, cell senescence and DNA damage were largely not involved in this process (Additional file 4: Figure S3A–F). Interestingly, our FACS analysis of DNA contents stained by Propidium Iodide (PI) revealed that overexpression ZNF24 significantly led A549i and Hop62i cells to be arrested in S phase (Fig. 2A, B). Cell proliferation was dramatically inhibited as assayed by EdU incorporation (Fig. 2C, D and Additional file 4: Figure S3G–H).

Fluorescent stain Carboxy Fluoroscein Succinimidyl Ester (CFSE) labels cell and each cell division dilutes its intensity of labeled cells by half. Thus, CFSE staining enables the visualization of eight or more generations of proliferating cells through FACS analysis. In this way, we found that ZNF24 overexpression significantly inhibited cell division (Fig. 2E–H). Reverse transcription-quantitative PCR (RT-qPCR) revealed that overexpression of ZNF24 inhibited expression of *CDK2*, *Cyclin D1* and *Cyclin E1* in A549i and Hop62i cells (Fig. 2I-J). Conversely, inhibition of ZNF24 up-regulated their expression in EKVX and H322 cells (Additional file 4: Figure S3I). Expression of these genes at protein level was further confirmed through Western blotting analysis (Fig. 2K–N). Overall, our results demonstrated that ZNF24 exerted tumor suppressive function through inhibiting cell cycle in lung cancer cells.

### ZNF24 induces cell cycle arrest through down-regulation of NF-κB signaling

**Fig. 3:**
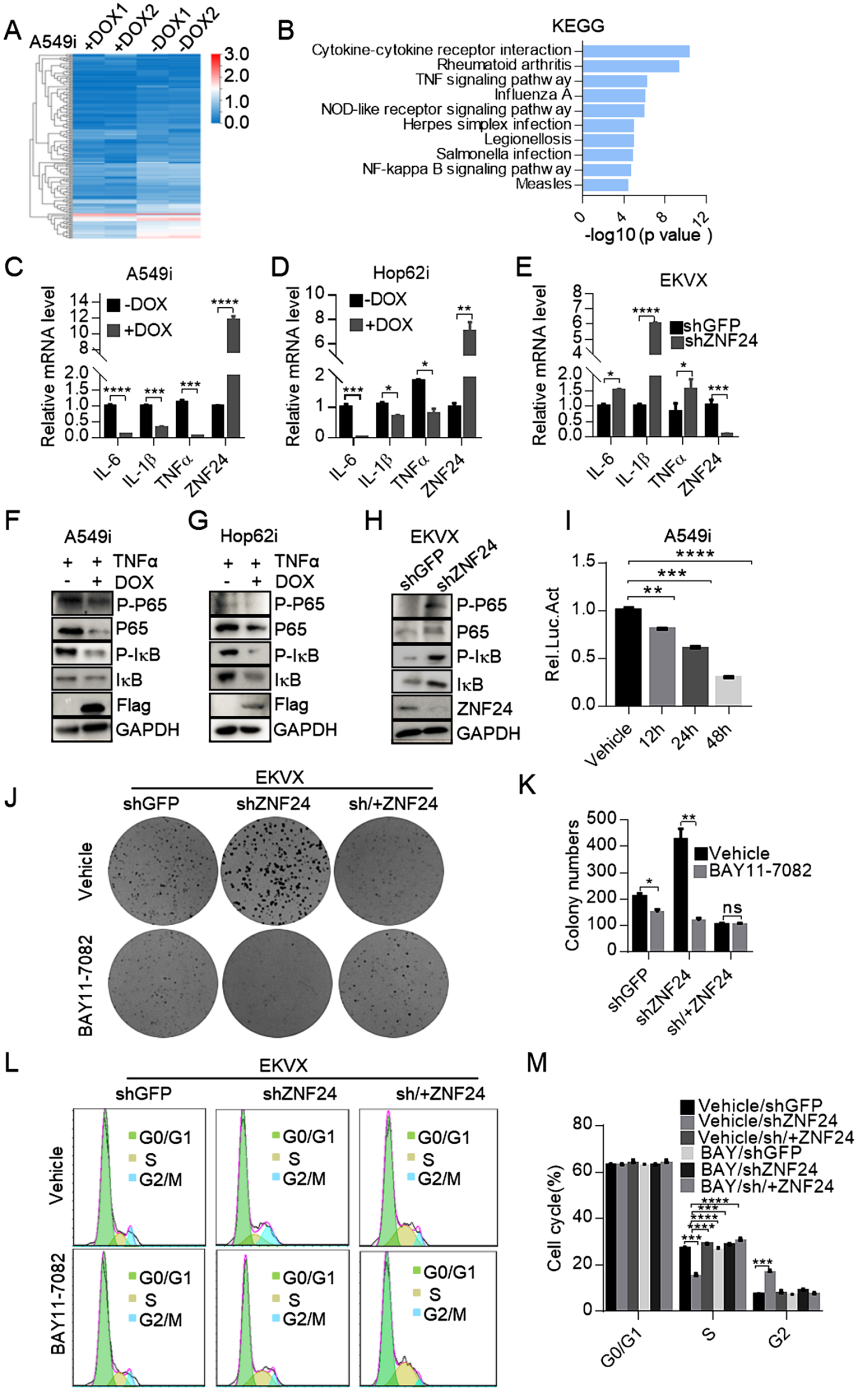
ZNF24 induces cell cycle arrest through NF-κB signaling pathways. **A** Heatmap representation of impact of ZNF24 expression on transcriptome of A549i cells. A549i cells were treated with 1 μg /mL of Dox for 48 h. RNAs were isolated and subjected to RNA-sequencing. Alteration in expression level were presented in Heatmap. **B** KEGG analysis of upregulated genes in A549 cell in response to ZNF24 knockdown. **C**, **D** and **E** RT-qPCR analysis of impact of ZNF24 on expression of NF-κB target genes. C. A549i was treated with Dox (1 μg/mL) for 48 h. *IL-6*, *IL-1β*, *TNFα* expression was quantified through RT-qPCR analysis. D. data on Hop62i cells. E. RNA was extracted from EKVX-shZNF24 cells (EKVX-shGFP cell served as control). Expression of the *IL-6*, *IL-1*β, *TNFα* was quantified through RT-qPCR. **F**, **G** and **H** Western blot analysis of impact of ZNF24 expression on NF-κB signaling pathway. F. A549i cells were treated with Dox (1 μg/mL) for 48 h. The cells were harvested for immunoblot analysis with indicated antibodies. G. data on Hop62i cells. H. data on EKVX-shZNF24. **I** Impact of ZNF24 expression on NF-κB reporter in A549i cells. A549i cells were transfected with the NF-κB reporter. Luciferase assays were performed 12, 24 and 48 h after Dox (1 μg/mL) administration. **J** Knock-down of ZNF24 sensitized EKVX cells to NF-κB inhibition. EKVX-shGFP, EKVX-shZNF24 and EKVX-sh/ + ZNF24 cells (1000) seeded in six-well plates, and treated with DMSO or NF-κB inhibitor (BAY11-7082, 2 μM) for 2 weeks. Cells were fixed and stained with 0.5% crystal violet in methyl alcohol. **K** Statistics of (**J**). **L** Impact of NF-κB inhibitor on cell cycling. EKVX-shGFP, EKVX-shZNF24 and EKVX-sh/ + ZNF24 cells seeded in six-well plates and treated with DMSO or NF-κB inhibitor (BAY11-7082, 2 μM) for 48 h. Cells were stained with propidium iodide (PI) and analyzed through FACS. **M** Statistics of **L**. Bars are represented as means ± SEM of the indicated number (n) of repeats. *P < 0.05, **P < 0.01, and ***P < 0.001 by Student’s t-test

In order to investigate how ZNF24 impact cell cycling, we compared gene expression profiles of A549i cells overexpressing ZNF24 (+ Dox) and control A549i cells (− Dox). RNA-seq analysis identified that 476 genes were significantly up-regulated (> twofold change) and 716 genes were down-regulated (< 0.5-fold change) (Fig. 3A and Additional file 4: Figure S4A). NF-κB signaling pathway caught our attention in KEGG analysis of pathways significantly under-represented in ZNF24 overexpressed lung cancer cells (Fig. 3B), as this pathway has been extensively reported to be involved in cell cycling [30–32]. Importantly, ZNF24 overexpression significantly inhibited transcription of target genes of NF-κB pathway, including *IL-1β*, *IL-6* and *TNFα* in A549i and Hop62i cells (Fig. 3C, D), while its knockdown significantly activated their expression in EKVX cells (Fig. 3E).

Furthermore, overexpression of ZNF24 significantly inhibited, while knockdown significantly increased, the phosphorylation level of P65 and IκB in lung cancer cells in response to TNFα stimulation (Fig. 3F–H). This alteration of phosphorylation of NF-κB signaling components are consequential as revealed by reporter assay (Fig. 3I and Additional file 4: Figure S4B). Taken together, our data indicated that ZNF24 effectively attenuated NF-κB activation by inhibiting P65 and IκB activation.

BAY11-7082 decreases NF-κB signaling and cell cycling through selectively and irreversibly inhibits phosphorylation of IκB [33, 34]. We found that BAY11-7082 dramatically suppressed colony formation capacity in EKVX-shZNF24 and H322-shZNF24 cells (Fig. 3J, K and Additional file 4: Figure S4C, D). We next assessed the effects of BAY11-7082 on cell cycle in ZNF24 knockdown cells. Results demonstrated an increased accumulation of S phase in BAY11-7082 treated cells compared to vehicle treated cells (Fig. 3L, M). Meanwhile, BAY11-7082 significantly inhibited the *CDK2* and *Cyclin D1*, *Cyclin E1* and *IL-1β*, *IL-6* and *TNFα* expression in ZNF24 knocked down cells (Additional file 4: Figure S4E–H).

Taken together, our data strongly argued that *ZNF24* inhibited NF-κB signaling in lung cancer cells.

### ZNF24 binds P65 promoter to negatively regulate its expression

NF-κB family is composed of five structurally related members, including NF-κB1 (also named P50), NF-κB2 (also named P52), RelA (also named P65), RelB and c-Rel, which mediates transcription of target genes by binding to a specific DNA element, κB enhancer, as various hetero- or homo-dimers [35].

**Fig. 4:**
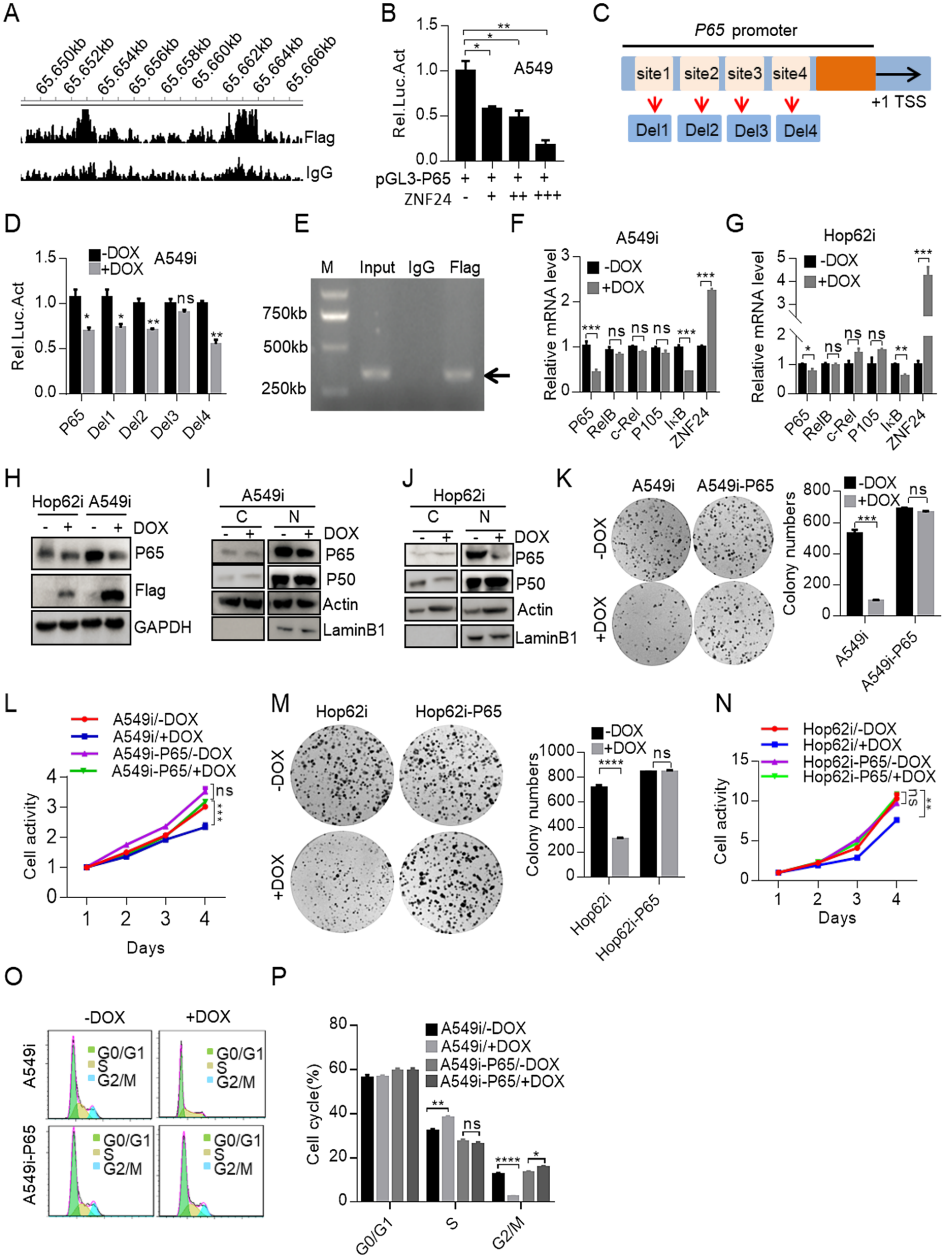
ZNF24 binds *P65* promoter to negatively regulate its expression. **A** Genome-wide analysis of ZNF24 binding sites in lung cancer cells through ChIP-seq analysis. A549i cells were treated with Dox (1 μg/mL) for 48 h. ChIP-seq was performed on DNA samples enriched for the Flag antibody. **B** Impact of ZNF24 expression on activity of *P65* promoter. *P65* promoter region was cloned into pGL3-Luciferase plasmid (designated pGL3-P65-Luciferase). The construct (0.5 μg) was transfected into A549 cells together with increasing amounts of plasmid (0.1, 0.2, 0.5 mg) for ZNF24 expression. Luciferase activity was monitored 24 h later. **C** Schematic diagram of 4 potential ZNF24-binding sites in *P65* promoter region. **D** ZNF24 binds to motif 3 to inhibit the activity of *P65*. Deletion mutants were generated as shown in C. Each of the mutant plasmids was respectively transfected into A549i cells by 1 μg/mL of Dox for ZNF24 expression. Luciferase was measured 24 h later. **E** ZNF24 bound to motif 3 of *P65* promoter region. A549i were treated with Dox (1 μg/mL) treatment. The Flag antibody was used to precipitate ZNF24 and DNA was extracted from the precipitants. Primers flanking 295 bp region of motif 3 was used to amplify DNA. **F**, **G** Impact of ZNF24 expression on *P65* mRNA expression level. A549i and Hop62i cells were treated with Dox (1 μg/mL) for 48 h. RNA was extracted for quantification of expression of designated genes through RT-qPCR. **H** Impact of ZNF24 expression on P65 expression in A549i and Hop62i cells. A549i and Hop62i cells were treated with Dox (1 μg/mL) for 48 h. The whole lysates were analyzed by western blot with the indicated antibodies. **I-J** Western blot evaluating the impact of ZNF24 on cellular localization of P65 and P50 proteins. Cytoplasmic and nuclear fractions were separated from Dox (1 μg/mL) treated A549i and Hop62i cells for 48 h, followed by western blot with indicated antibodies. **K** Impact of P65 expression on the colony forming ability of A549i cells. A549i cells and A549i-P65 (1000 cells) were seeded in 6-well-plates with media supplemented with Dox (1 μg/mL) for 2 weeks. Left: representative pictures. Right: statistics of colony number. A549i-P65 stands for ectopic expression of P65 in A549i cells. **L** Impact of P65 expression on the growth rate of A549i. A549i and A549i-P65 (1000 cells) were seeded in 96-well-plates and treated with Dox (1 μg/mL) for 4 days. Cell growth was measured by CCK8. **M** Impact of P65 expression on the colony forming ability in Hop62i cells. Hop62i cells and Hop62i-P65 (1000 cells) were seeded in 6-well-plates by Dox (1 μg/mL) for 2 weeks before quantification for colonies and statistics of colony number. Left: representative pictures. Right: statistics of colony number. Hop62i-P65 for re-overexpression P65 in Hop62i cells. **N** Impact of P65 expression on the growth rate of ability in Hop62i, Hop62i-P65 cells. Hop62i, Hop62i-P65 (1000 cells) were seeded in 96-well-plates and treated with or without Dox for 4 days. Cell growth was detected by CCK8. **O** Impact of P65 expression on cell cycle in A549i cells. A549i and A549i-P65 cells treated with Dox (1 μg/mL) for 48 h, followed by staining with propidium iodide (PI). Cell cycle is analyzed by flow cytometry. **P** Statistics of **O**. Bars are represented as means ± SEM of the indicated number (n) of repeats. *P < 0.05, **P < 0.01, and ***P < 0.001 by Student’s t-test

To elucidate the mechanism underlying ZNF24’s capacity to inhibit NF-κB signaling activity, we conducted ChIP-seq on ZNF24 overexpressing A549 cells. We found that ZNF24 bound 27,076 genomic sites, including the promoter region of *VEGFA* and *PDGFRB* genes previously reported to be ZNF24 targets [9–11], suggesting that our ChIP was successful (Additional file 4: Figure S5A, B). Interestingly, we found that that ZNF24 precipitated the promoter region of the *P65* (Fig. 4A). ZNF24 inhibited *P65* promoter activity in lung cancer cells in a dose and time-dependent manner as revealed in luciferase reporter assay (Fig. 4B and Additional file 4: Figure S5C). JASPAR (https://jaspar.genereg.net/) predicted four possible ZNF24-binding sites in *P65* promoter region (Fig. 4C). To pin down which site mediated ZNF24’s suppressive role, we generated truncation mutations to check the impact of deletion of individual site on promoter activity in lung cancer cells overexpression ZNF24. Our results suggest that deletion of motif 3, with sequence of 5ʹ-CACTAATTCTGTCAT-3ʹ, dramatically impaired the ability of ZNF24 to inhibit *P65* promoter activity in lung cancer cells (Fig. 4D). Importantly, we were able to confirm the ability of ZNF24 to bind this site through ChIP-PCR (Fig. 4E). Collectively, these results indicated that ZNF24 bound *P65* promoter to negatively regulate the P65 expression.

Furthermore, RT-qPCR analysis revealed overexpression of ZNF24 inhibited *P65* transcription (Fig. 4F, G). Inhibition of P65 expression was further confirmed at protein level (Fig. 4H). Notably, transcription of *IκB* gene was also downregulated by ZNF24 (Fig. 4F, G), suggesting ZNF24 affected multiple signaling elements of NF-κB pathway. We also found that ZNF24 expression decreased the abundance of P65 protein both in cytoplasmic and nuclear fractions (Fig. 4I-J). Our data revealed that ZNF24 inhibited the expression of P65 in lung cancer cells.

Importantly, ectopic expression of *P65* gene in ZNF24 overexpressing cells rescued colony formation in 2-D plates and proliferation of lung cancer cells (Fig. 4K–N). Meanwhile, ectopic expression of P65 also rescued S phase blockage and expression of cell cycle-related genes including *CDK2*, *Cyclin D1* and *Cyclin E1*, in addition to NF-κB target genes including *IL-1β*, *IL-6* and *TNFα* in lung cancer cells overexpressing ZNF24 (Fig. 4O, P and Additional file 4: Figure S5D–G). Collectively, our results indicated that ZNF24 bound to *P65* promoter and negatively regulated its expression to impact cell cycling and growth.

### Combinational inhibition of KRAS, NF-κB and PD-1 effectively shrinks Kras^G12D^/ZNF24^−/−^ lung cancers

**Fig. 5:**
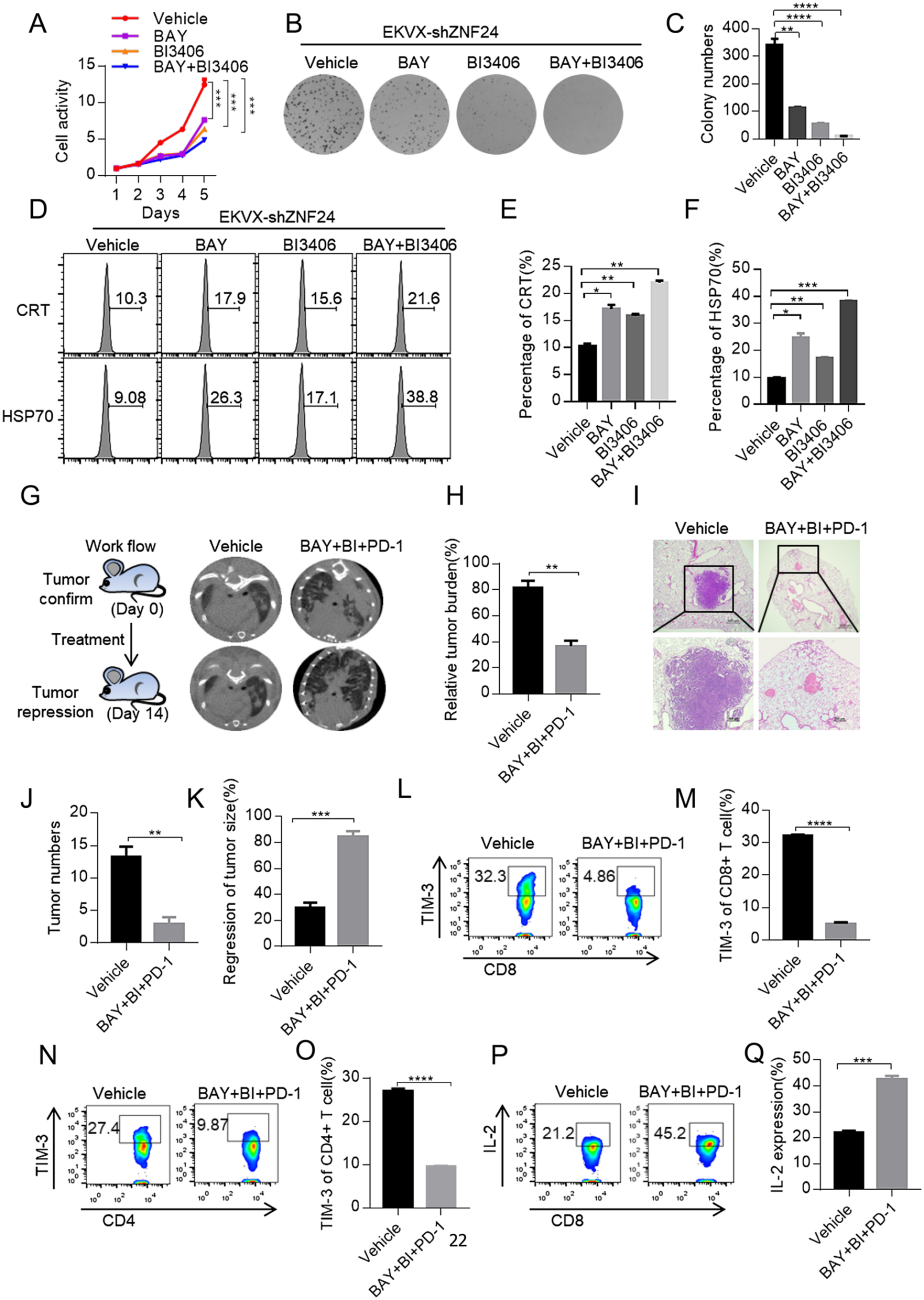
Combinational inhibition of KRAS, NF-κB and PD-1 effectively shrinks Kras^G12D^/ZNF24^−/−^ lung cancers. **A** Combination of NF-κB inhibitor (BAY11-7082) and/or KRAS inhibitor (BI3406) effectively inhibited proliferation of ZNF24 knockdown EKVX cells. EKVX-shZNF24 (1000 cells) were seeded in 96-well plates. Cells were treated with BAY (2 μM) and/or BI3406 (1 μM) for 5 days. Cell viability was checked with CCK8 after drug treatment. **B** NF-κB inhibitor (BAY11-7082) and/or KRAS inhibitor (BI3406) effectively inhibited colony forming ability of ZNF24 knockdown EKVX cells. EKVX-shZNF24 (1000 cells) seeded in 6-well plates. Cells were treated with BAY (2 μM) and/or BI3406 (1 μM) for 2 weeks before colony quantification. **C** Statistics of colony number of **(B)**. **D** Induction of immunogenic cell death by NF-κB inhibitor (BAY11-7082) and/or KRAS inhibitor (BI3406) in EKVX-shZNF24 cells. EKVX-shZNF24 cells were treated with BAY (2 μM) and/or BI3406 (1 μM) for 24 h. Expression of CRT and HSP70 was determined by flow cytometry. **E**, **F** Statistics of **(D)**. **G** Combinational treatment with BAY11-7082, BI3406 and PD-1 antibody (designated BAY + BI + PD-1) effectively shrinks lung tumor in K-sgZNF24 mice. Lsl-Kras^G12D^ mice were treated with the recombinant lenti-virus co-expressed Cre and CRISPR/Cas9 by nasal drip. Mice were treated with BAY11-7082 (20 mg/kg/day, intraperitoneal), BI3406 (25 mg/kg/day, gavage) and PD-1 antibody (5 mg/kg, every other day, intraperitoneal). Tumor burdens were documented with CT. **H** Quantification of tumor burden of combinationally treated mice of **(G)**. **I** Representative images of Hematoxylin and eosin (H&E) stained lung tissues obtained from different treatment groups. **J** Quantification of tumor numbers of mice of **(G)**. **K** Relative tumor volume of mice of **(G)**. **L–O** Combinational treatment with BAY11-7082, BI3406 and PD-1 antibody (designated BAY + BI + PD-1) activated T cell mediated immunity in tumor microenvironment. Expression of TIM-3 of tumor-infiltrating CD8 + T cells and CD4 + T cells were determined by flow cytometry. **P, Q** Combinational treatment with BAY11-7082, BI3406 and PD-1 antibody (designated BAY + BI + PD-1) activated expression of effector cytokine in tumor infiltrating CD8 + T cells. Tumor-infiltrating CD8 + T cells of **(G)** were intracellularly stained for FACS analysis of expression of IL-2. Data are means ± SEM of three independent experiments. *p < 0.05, **p < 0.01, ***p < 0.001 (student’s t-test)

We then asked the potential implication of ZNF24’s tumor suppressive role in treating lung cancer patients. Supper activation of KRAS remains a significant obstacle for successful lung cancer treatment in clinic. We found that around 41.5% of *KRAS* mutation positive lung cancer patients harbor low expression of ZNF24 (designated Zno/K* patients, Additional file 2: Table S2). We then tested whether NF-κB inhibitor synergized KRAS inhibitors in treating Zno/K* patients on EKVX-shZNF24, a lung cancer cell line harboring wildtype (WT) but super active KRAS [36]. For this purpose, we tested BI3406, a pan-KRAS inhibitor capable of selectively blocking SOS1–KRAS interaction [37]. We found that combination of BAY11-7082 and BI3406 effectively reduced proliferation and colony formation in 2-D plates of EKVX-shZNF24 in stark contrast to the limited efficacy seen in mono-therapy (Fig. 5A–C). Moreover, this combinational therapy also effectively shrank the xenograft tumor on nude mice derived from this cell line grown (Additional file 4: Figure S6A–C).

To test the treatment effect of this combination scheme on transgenic lung cancer mouse model for better mimicking patients in clinic, we generated a cohort of K-sgZNF24 mice as shown in Fig. 1M to model autochthonous lung cancer formation in immune competent hosts. Surprisingly, we found that the combinational inhibition with BAY11-7082 and BI3406 exhibited limited efficacy (Additional file 4: Figure S6D–H), suggesting tumors grown in vivo in the immunocompetent hosts behaved differently from cells grown on 2-D plates and xenografted tumors in nude mice. In order to further confirm the impact of host immunity on therapeutic effect of BAY11-7082 and BI3406, we generated ZNF24 knockdown LLC (LLC-shZNF24), a murine lung cancer cell line of C57BL/6J background. We then inoculated LLC-shZNF24 cells into nude mice or C57BL/6J mice for growing allograft tumors in immune deficient or competent hosts. We found that for tumors in nude mice, singlet treatment with BAY-11–7082 or BI3406 resulted in tumor shrinkage and that combinational treatment resulted in even greater shrinkage (Additional file 4: Figure S6I–K). In contrast, for tumors in immunocompetent C57BL/6J mice, the tumor shrinkage is drastically compromised with either singlet or combo (Additional file 4: Figure S6L–N). The treatment effect was largely not seen in none of the treatment groups on LLC-shZNF24/shP65 derived tumors in C57BL/6J mice (Additional file 4: Figure S7A–C). As NF-κB transcribes many cytokine genes, which play important role in shaping the local tumor microenvironment and could potentially impact on treatment effects of our above treatment schemes. Indeed, we found that expression of *IL-1β*, *IL-6* and *TNFα* was dramatically reduced in lung cancer cells in response to BAY11-7082 treatment (Additional file 4: Figure S7D). Taken together, combinational treatment with BAY-11–7082 and BI3406 is not effective to shrink Zno/K* tumors in immunocompetent hosts.

We, therefore, went on to find an effective treatment strategy for K-sgZNF24 lung cancers.

Previous studies observed inconsistent results on whether NF-κB signaling in cancer cells dampens host’s anti-tumor immune response [38–40]. However, it’s been solid that NF-κB is an important transcription factor for PD-L1 expression in lung cancer cells [41–43]. We found that treatment with BAY11-7082 significantly decreased the expression of PD-L1 in A549 cells (Additional file 4: Figure S7E). Interestingly, we found that BAY11-7082 treatment resulted in immunogenic cell death (ICD) as revealed by overexpression of Calreticulin, HSP70, HMGB1 and ATP in treated cells [44] (Fig. 5D–F and Additional file 4: Figure S7F, G), suggesting that BAY11-7082 compound could activate antigen presenting cells and immunologically synergized with KRAS inhibitors for treating K-sgZNF24 lung cancers [45].

We then generated a cohort of mice bearing K-sgZNF24 lung cancers as shown in Fig. 1M and treated these mice with BAY11-7082, BI3406, and anti-mouse PD-1 antibody for 2 weeks (Fig. 5G). Micro-CT imaging revealed almost complete regression of lung tumor (Fig. 5G, H). Pathological analysis lung sections revealed many residual tumor nodules featuring thickened alveolar wall and fibrosis, indicative of the drastic healing process (Fig. 5I–K). Meanwhile, we found that there was no obvious treatment effect with anti-mouse PD-1 antibody alone in K-sgZNF24 mice (Additional file 4: Figure S7H–J).

We checked the impact of expression level of ZNF24 on infiltration of CD4 + and CD8 + T cells into tumors through FACS analysis. We found that tumor-infiltrating CD4 + T cells were comparable between Kras^G12D^-sgControl tumors and Kras^G12D^-sgZNF24 tumors (Additional file 4: Figure S8A). In contrast, the amount of tumor-infiltrating CD8 + T cell were lower in Kras^G12D^-sgZNF24 tumors than in Kras^G12D^-sgControl tumors (Additional file 4: Figure S8B). This result suggested that CD8 + T cells may play a bigger role in inhibiting growth of Kras^G12D^-sgZNF24 tumor. We checked the role of CD4 + and CD8 + T cells during treatment. We found our antibodies effectively depleted CD8 + or CD4 + T cells (Additional file 4: Figure S8C, D). Data showed that depletion of either CD4 + or CD8 + T cells significantly inhibited the effect of combination therapy to shrink tumors (Additional file 4: Figure S8E, F) and that both CD4 + and CD8 + T cells playing critical roles in shrinking tumors during combinational treatment*.* Furthermore, FACS analysis revealed that combinational treatment didn’t significantly change the amount of tumor-infiltrating CD8^+^ and CD4^+^ T cell (Additional file 4: Figure S8G–I). Instead, this treatment rescued exhaustion of these T cells, as suggested by decreased TIM-3 expression (Fig. 5L–O). In line with this, FACS analysis of expression of IL-2, IFNγ and TNF-α expression in T cells through intracellular staining revealed that combination therapy resulted in increased effector function of tumor-infiltrating CD8 + T cells (Fig. 5P, Q and Additional file 4: Figure S8J, K). Taken together, these data indicate that combinational inhibition of NF-κB, KRAS and PD1 are effective to treat Kras^G12D^/ZNF24^−/−^ lung cancers.

### ZNF24-NF-κB signaling axis is clinically relevant

**Fig. 6:**
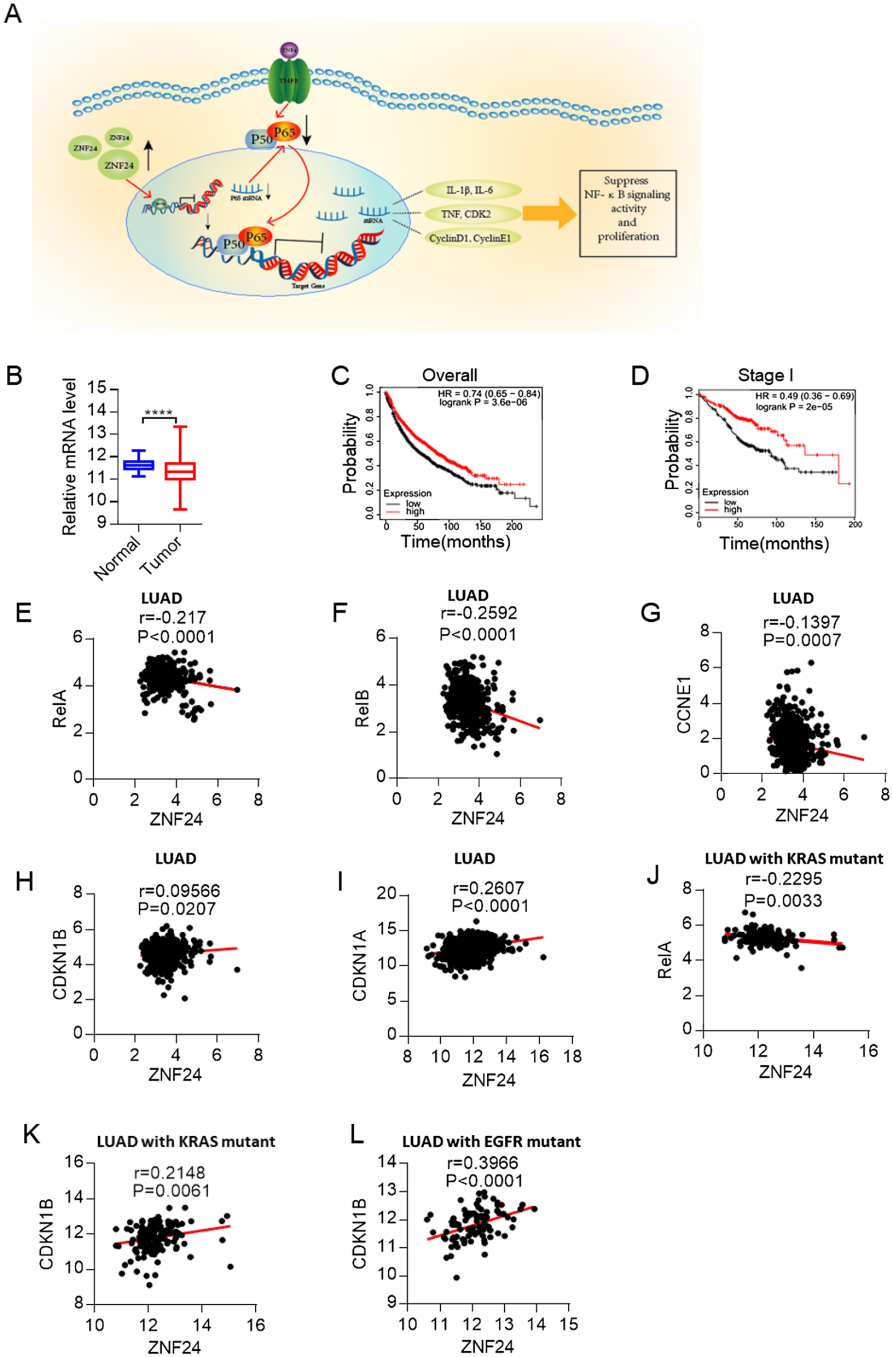
ZNF24-NF-κB signaling axis is clinically relevant. **A** Schematic model of ZNF24-P65-NF-κB signaling axis in lung cancer. **B** Expression of *ZNF24* mRNA in lung cancer tissue and para-tumoral tissues of patients with TCGA (http://xena.ucsc.edu/). **C** K–M survival of lung cancer patient (all stage, n = 1927) (http://kmplot.com). **D** K–M survival of lung cancer patient (Stage I, n = 449) (http://kmplot.com). **E**–**F** Correlation between expression of *ZNF24* and that of *RelA* or *RelB*. Expression data of lung adenocarcinoma cancer patients from TCGA database (analyzed through UCSC Xena). **G**, **H** and **I** Correlation between expression of *ZNF24* and that of *CCNE1*, *CDKN1A* or *CDKN1B*. Expression data of lung adenocarcinoma cancer patients from TCGA database (analyzed through UCSC Xena). **J**, **K** Correlation between expression of *ZNF24* and that of *RelA* or *CDKN1B*. Expression data of lung adenocarcinoma cancer patients positive for *KRAS* mutation from TCGA database (analyzed through UCSC Xena). **L** Correlation analysis of expression between *ZNF24* and *CDKN1B*. Expression data of lung adenocarcinoma cancer patients positive for *EGFR* mutation from TCGA database (analyzed through UCSC Xena)

Our data so far showed that *ZNF24* inhibited NF-κB signaling activity to function as a lung cancer suppressor (see our model in Fig. 6A). Further supporting *ZNF24* as a lung cancer suppress gene, analysis of TCGA data revealed lower ZNF24 mRNA level in primary lung tumor in comparison to para-tumoral lung tissues (Fig. 6B). Moreover, higher expression level of ZNF24 in lung adenocarcinoma was significantly correlated with longer survival of patients in all stages (Fig. 6C). Of note, this trend was also found in stage I patients (Fig. 6D), strongly suggesting *ZNF24* as a clinically relevant TSG.

We then went on to investigate the clinical relevance of our finding that *ZNF24* exerted its TSG function through inhibiting NF-κB signaling. In line with our model shown in Fig. 6A, analysis of TCGA database with UCSC Xena (http://xena.ucsc.edu/) revealed significant reverse correlation between expression level of *ZNF24* and those of *RelA* (*P65*) and *RelB* respectively (Fig. 6E, F). We also detected significant reverse correlation between *ZNF24* and *CCNE1* (encoding Cyclin E1) (Fig. 6G). Interestingly, we detected positive correlations between *ZNF24* and Cyclin-dependent kinase inhibitors including *CDKN1A* and *CDKN1B* (Fig. 6H, I). These data strongly suggest that negative regulation of NF-κB signaling by *ZNF24* is clinically relevant in lung cancer patients.

A significant portion (41.5%) of *KRAS* mutation positive lung cancer patients harbor low expression of ZNF24. Likewise, we found that expression level of *ZNF24* is significantly reverse-correlated with that of *RelA* and positively correlated with that of *CDKN1B* in these *KRAS* mutation positive patients (Fig. 6J, K). Moreover, A significant portion (51.8%) of *EGFR* mutation positive lung cancer patients harbor low expression of ZNF24 (Additional file 3: Table S3). We detected positive correlations between *ZNF24* and *CDKN1B* in *EGFR* mutation positive patients (Fig. 6L).

We have shown that *ZNF24* negatively regulates expression of PD-L1 in lung cancer through inhibiting NF-κB activity. We then analyzed the expression of ZNF24, P65, and PD-L1 in human lung cancer samples through IHC (Additional file 4: Figure S9A). In line with our conclusion, we found that ZNF24 is inversely correlated with P65 and PD-L1 in lung cancer. We also found positive correlations between P65 and PD-L1 (Additional file 4: Figure S9B–D). Taken together, mechanism underlying ZNF24 in lung cancer suppressive role revealed in our current study is clinically relevant.

## Discussion

Here, we identified *ZNF24* as a novel potent tumor suppressor gene in lung cancer through genome-wide knockout screening. Ectopic expression of ZNF24 arrested lung cancer cells in S phase. Mechanistically, we found that ZNF24 inhibited the activation of NF-κB signaling pathway through binding the promoter region of *P65*, whereby inhibiting its transcription. We found that a significant portion of *KRAS* mutation positive lung cancer patients express low level of ZNF24 (designated Zno/K* patients). Using transgenic mouse model of lung cancer, we showed that Zno/K* lung cancers were sensitive to combinational therapy with NF-κB inhibitor, KRAS inhibitor and PD-1 antibody.

ZNF family members have been reported to specifically bind to chromosomal sites containing TCAT repeat and thus activate (like *β-Catenin* and *Wnt7B* [46, 47]) or inhibit (like *VEGF* and *PDGFRB* [9–11]) target genes transcription. It will be interesting to fully characterize the transcriptional complexes organized by ZNF24 to elucidate the environmentally dependent inhibition or activation of target gene expression. The N-terminal SCAN domain of ZNF24 has been reported to be involved in interaction with a lot of partners, including protein, DNA and RNA [7]. Our current work clearly showed that ZNF24 inhibited transcription of *P65* gene in lung cancer cells. However, Le et.al. found that ZNF24 activated transcription of *P65* gene in oral mucositis model [48]. Elucidating the mechanisms underlying environment-dependent selection of binding partners is not only an intriguing project, but could potentially be translated into clinical therapies.

The impact of NF-κB in cancer cells on host’s immune surveillance has been controversial, with some showing promoting [39] and others repressing [40]. We showed in this work that loss of function of *ZNF24* in lung cancer cells activated NF-κB signaling, thus creating the Achilles’ heel for targeting with NF-κB inhibitors. The important role played by NF-κB in both innate and acquired immune cells [49] could reconcile the inconsistency of the impact of NF-κB inhibitor on cancer cells in vitro versus tumors in vivo.

*KRAS* mutation positive lung cancer patients remains a huge challenge in clinic. Currently, G12C mutant *KRAS* selective inhibitors have hit market [50]. However, drug resistance to these inhibitors severely limited the success in lung cancer clinic [51]. Moreover, G12V and G12D mutation represents higher frequency in lung cancer patients [52, 53]. We have found in current work that almost half of the *KRAS* mutation positive lung cancer patients express low level of ZNF24. More importantly, we developed the precision treatment scheme on transgenic mouse model of autochthonous lung cancer: Combination of pan-KRAS inhibitor, NF-κB inhibitor and PD-1 inhibitor effectively shrank *KRAS* mutation positive/ZNF24-low lung cancers. Although many more questions remain to be answered, including how immunogenic cancer cell death contributes to treatment effect; how T cells are activated; and how DCs cross-talks with tumor cell and T cells, the precision medicine we put forward in current work for *KRAS* mutation positive/ZNF24-low lung cancer patients warrant further study.

In our study, we have shown that host immunity plays a critical role in shaping the effectiveness of a particular treatment scheme. Echoing this effect, recent years have seen rapid development of immune checkpoint blockade (ICB)-based immunotherapy. However, benefit of ICB treatment is limited to a minor portion of patients. Auxiliary treatment may be needed to improve efficacy of ICB treatment. Chemotherapy and targeted therapy improved the immunogenicity of tumor cells and improved CD8 + T cell infiltration or inhibit immune inhibitory cells in tumor microenvironment [54, 55]. Adoptive transfer of mesenchymal stromal cells (MSCs) equipped by IFNα has been reported to achieve anti-tumor effect by improving the function of CD8 + T cells in the tumor microenvironment [56]. This point is embodied in our study that combinational inhibition of KRAS and NF-κB improved immunogenicity of tumor cells, recruiting effector T cells and thus synergized with PD-1 inhibitors to shrink *KRAS* mutation positive lung cancer of low ZNF24 expression.

## Conclusions

Our study described a genome-wide screening of lung cancer suppressor genes in vivo in mice. Combining our screening results and TCGA data, we found *ZNF24* as a novel, potent and clinically relevant TSG of lung cancer. *ZNF24* exerts it lung tumor suppressive function through inducing cell cycle arrest in S phase. We further defined its molecular mechanism: ZNF24 bound the promoter region of *P65* and inhibited its transcription; *ZNF24* thus inhibited signaling activity of NF-κB pathway and the expression related cyclin/CDKs. ZNF24-P65-cyclin/CDK axis was clinically relevant. We also found that *ZNF24* was absent in a significant portion of lung cancer patients. Using mouse model recapitulating lung cancers Kras^G12D^/ZNF24^−/−^ patients, we found that combinational inhibition of KRAS, NF-κB and PD-1 effectively shrank the lung tumor.

## Materials and methods

### Animal care and use

All mice were raised in a specific pathogen-free environment in the Jinan University and all experimental protocols were approved by the Jinan University. All animal work is carried out in accordance with the approved protocols. In the single-dose virus infusion experiment, the siblings of double transgenic mice were randomly divided into two groups (3–4 in each group). TetO-Kras^G12D^/CC10rtTA (designated KC) double transgenic mice were fed with doxycycline (Dox)-containing diet for 2 months to induce lung Adenocarcinoma. Retrovirus for overexpressing ZNF24 (KC-Z) or Control (KC-C) was inhaled through the nostrils into lungs. After recovery for a week, all the mice were fed with diet containing Dox until the day of the sacrifice. The lung tissues were stained with hematoxylin eosin. Computed Tomography (CT, PINGSENG Healthcare) was used to quantify tumor burdens. To quantify the tumor burden, we calculated the total size (mm^3^) of all tumor regions in H&E sections under the microscope.

Lsl- Kras^G12D^ Mice of 6–8 weeks of age were treated with recombinant lenti-virus co-expressing Cre and CRISPR/Cas9 via nasal inhalation. Tumor burdens were monitored through CT in mice with ZNF24 knockout (K-sgZNF24 mice) and with Td-Tomato knockout (K-sgTD mice for control) six months after infection.

### Mouse treatment

The sgRNA targeting ZNF24 was cloned into pSECC plasmid. The resultant plasmid was sequenced to verify before used to prepare recombinant viruses. These viruses were dripped into Lsl-Kras^G12D^ mice through nostrils. Tumor burden was documented by computed tomography (CT, PINGSENG Healthcare) six months after lung cancer induction. Mice were treated with NF-κB inhibitor BAY11-7082 (selleckchem, 20 mg/kg/day), BI3406 (selleckem, 25 mg/kg/day) and anti-PD-1 (BioXcell, BP0033-2, 5 mg/kg). Tumor burden were monitored by computed tomography.

### In vivo xenograft model

8-week-old male BALB/C Nude mice were used for xenotransplantation study (Guangdong Medical Lab Animal Center). Briefly, 2 × 10^6^ A549i cells were inoculated subcutaneously on the right back of mice in 100 μL Matrix (Gel356237, CORNING). Mice were randomly grouped to give doxycycline or normal diet when tumor volume reached about 100 mm^3^. Body weight and tumor volume (D × d^2^/2 (mm^3^)) were measured every 2 days (D was the longest and d was the shortest diameter). At the end of treatment, tumors were harvested for photographing and weighing.

### Cell culture and generation of engineered cell lines

HEK293T, NCI-H446, EKVX, NCI-H322, NCI-H460, NCI-H1975, PC9, HCC827, Hop62, NCI-H3255, Hop92 and A549 were purchased from ATCC as part of NCI-60 (American Typical Culture Collection, Manassas, VA, USA). BEAS-2B, and HEK293T cells were provided by Dr. Kwok-Kin Wong (The Helen and Martin Kimmel Center for Stem Cell Biology, NYU). All cell lines were held in a standard tissue incubator at 37° C with 5% CO_2_. BEAS-2B, HEK293 cells were cultured in DMEM supplemented with 10% FBS and 1% penicillin /glutamine. (FBS, Gibco, Life Technologies, Carlsbad, CA, USA). The remaining lung cancer cells were cultured in RPMI-1640. For preparing recombinant lenti-virus, HEK293 cells were transfected with two packaging plasmids (psPAX2 and pMD2.G) together with a pLVX-TetOne-Puro vector control or the same vector constructs for expressing ZNF24, RelA respectively. Cells were changed with fresh medium after 6 h. The recombinant virus-containing medium was filtered with 0.22 µm and used to infect cells in the presence of polybrene (8 µg/mL), followed by selection with 1 μg/mL puromycin (J593, Amresco) for 1 week. For shRNA knockdown, cells were infected lenti-virus packaged from pLKO.1-shGFP-Puro vector control or the same vector constructs encoding shZNF24, shNR3C2, shCST4, shARHGDIG, shCRYBB3. Cells were selected the cells with 1 μg/mL of puromycin for 1 week.

### Reagents and antibodies

Following reagents were used in current study: Doxycycline hyclate (Dox, D9891, Sigma, St. Louis, MO, USA), Protease and phosphatase inhibitor cocktail (CatNO:C0001, C0002, TargetMol), trizol (15596018, ambion), Lipofectamine3000 (L3000015, Invitrogen), Western blotting substrate (WBKLS0500, Millipore), Cell Counting Kit-8 (TN623, DojindoLaboratories). Cell Signaling Senescence β-Galactosidase Staining Kit (#9860, CST), dual-specific luciferase assay kit (Promega), anti-ZNF24 (HPA024062, Sigma Aldrich), anti-ZNF24 (11219–1-AP, Proteinch), anti-Caspase1 (22915–1-AP, Proteinch), anti-P-MLKL (phospho S358, D6H3V, CST), anti-LaminB1 (ab16048, Abcam), anti-CDK2, Cyclin B1, Cyclin D1 (ab228528, Abcam), anti-Cyclin E1 (ab33911, Abcam), anti-Caspase9 (#9504, CST), anti-Caspase3 (#9661, CST), anti-Caspase8 (#8592, CST), anti-FLAG (#14793, CST), anti-β-actin (A5316, Sigma), anti-P65 (ab16502, Abcam), anti-P50 (ab32360, Abcam), anti-P-P65 (phospho S536, ab86299, Abcam), anti-IκBα (ab32518, Abcam), anti-p-IκBα ( phospho S36, ab133462, Abcam), anti-GAPDH (ab8245, Abcam).

The antibody in flow cytometry as follows: anti-mouse-CD45 (559664, BioLegend, APC), anti-mouse-CD8a (45–0081-82, eBioscience, PerCP-Cyanine5.5), anti-mouse-CD4 (48–0041-82, eBioscience, BV421), anti-mouse-TIM-3 (119703, BioLegend, PE), anti-mouse-TNF-α (506305, Biolegend PE), anti-Human-Calreticulin (D3E6, CST), anti-Human-HSP70 (ab2787, Abcam), anti-HMGB1 (10829–1-AP, Proteinch), anti-mouse-IL-2 (12–7021-82, eBioscience, PE), anti-mouse- IFNγ (12–7311-81, eBioscience, PE).

### Constructs

Plasmids, pLVX-TetOne-Puro and pLVX-TetOne-zeocin were purchased from Clontech, and psPAX2, pMD2.G and pLKO.1-puro from Addgene. plasmids encoding shRNAs were constructed by standard molecular biology techniques. shRNA target sequences were as follow:

shCST4: 5ʹ- CTTTCGAGATCTACGAAGTTC-3ʹ.

shARHGDIG: 5ʹ-CCCAGGAGTATGAGTTTGTGA-3ʹ.

shCRYBB3: 5ʹ- CCCAGGAGTATGAGTTTGTGA-3ʹ.

shZNF24: 5ʹ-GAGGATTTGGAGAGTGAACTT-3ʹ.

shNR3C2: 5ʹ-CCTTGCCTTCAGCAAGACAAT-3ʹ.

The constructs, pCDH-NF-κB reporter, FLAG-tagged ZNF24 and pGL3-P65-Luciferase were cloned by standard molecular biology techniques and verified through sequencing.

### Cell proliferation assay

Cell proliferation test: 1 × 10^3^ cells (A549i, Hop62i) were inoculated into each well of 96-well plates and cultured overnight. 1 μg/mL of Dox was added for 5 days in 1640 culture medium containing 10% FBS. The proliferative activity was then determined using the CCK8 following the manufacturer's protocols.

### Colony formation assay

1000 cells were seeded in 6-well plates containing medium supplemented with 10% FBS. The cells were cultured without or with Dox incubation for 14 days. Fixed in methyl alcohol and stained with 0.5% crystal violet.

### Soft-agar colony formation assay

The soft-agar colony formation test was carried out in soft agar (0.6% lower gel and 0.35% upper gel). Add 1 mL of 0.5% base gel to each well of the six-well plates and let stand for 3 h. 1 × 10^4^ A549i and Hop62i cells were mixed with the upper gel and Dox (1 μg/mL), and then seeded in 6-well plates. 0.5 mL 1 × fluid media containing Dox (1 μg/mL) was added weekly to the upper gel surface. The cells were cultured around 3–4 weeks with or without Dox treatments before imaging and colonies counting.

### Senescence-associated β-Galactosidase (SA-β-Gal) staining assay

SA-gal staining was performed with cell signal transduction aging galactosidase Staining Kit (CST, #9860). 200,000–300,000 cells were seeded in each well of the six well plates and cultured until staining time. A549i and Hop62i cells were treated with Dox (1 μg/mL) for 2 days, and then SA-gal staining was performed to observe the effect on senescence.

### Cell cycle analysis

A549i and Hop62i cells were treated with or without 1 μg/mL of Dox for 48 h, and fixed with 70% ethanol at − 20 °C overnight. After fixation, the cells were subsequently centrifuged at 3000 × g for 5 min and washed with PBS. Cell cycle was performed with the cell cycle kit (Beyotime, C1052) following the manufacturer’s instructions. Cell cycle distribution was analyzed using BD Accuri™ C6 flow cytometer.

### RNA extraction and real-time RT-PCR

Total RNA was extracted with Trizol reagent and analyzed by real-time PCR to detect the mRNA level of the gene. 2 μg of total RNA was reverse-transcribed to cDNA through AccuRT Genomic DNA Removal Kit (G492, Applied Biological Materials). Real-time PCR was performed with Bio-Rad Real-Time PCR System and SYBR Green qPCR Mix (110344, Monad). The data shown is the relative abundance of the referred mRNA normalized to Actin. The sequence of gene-specific Primers is as follows:

CDK2-Forward: 5′- ATGGATGCCTCTGCTCTCACTG-3′,

CDK2-Reverse: 5′- CCCGATGAGAATGGCAGAAAGC-3′,

Cyclin E1-Forward: 5′- TGTGTCCTGGATGTTGACTGCC-3′,

Cyclin E1-Reverse: 5′- CTCTATGTCGCACCACTGATACC-3′,

Cyclin D1-Forward: 5′- TCTACACCGACAACTCCATCCG-3′,

Cyclin D1-Reverse: 5′- TCTGGCATTTTGGAGAGGAAGTG-3′,

P65-Forward: 5′- TGAACCGAAACTCTGGCAGCTG-3′,

P65-Reverse: 5′- CATCAGCTTGCGAAAAGGAGCC-3′,

RelB-Forward: 5′- TGTGGTGAGGATCTGCTTCCAG-3′,

RelB-Reverse: 5′- TCGGCAAATCCGCAGCTCTGAT-3′,

C-Rel-Forward: 5′- AGTTGCGGAGACCTTCTGACCA-3′,

C-Rel- Reverse: 5′- CGTGATCCTGGCACAGTTTCTG-3′,

P105-Forward: 5′- GCAGCACTACTTCTTGACCACC-3′,

P105- Reverse: 5′- TCTGCTCCTGAGCATTGACGTC-3′,

Actin-Forward: 5′-ACGTGGACATCCGCAAAG-3′,

Actin- Reverse: 5′- GACTCGTCATACTCCTGCTTG-3′,

ZNF24-Forward: 5′- GTGACAGTGCTGGAGGATTTGG-3′,

ZNF24- Reverse 5′- GGTTCTCCACAGCATCAAGCTC-3′,

IL-6-Forward: 5′- AGACAGCCACTCACCTCTTCAG-3′

IL-6- Reverse: 5′-TTCTGCCAGTGCCTCTTTGCTG-3′.

IL-1β-Forward: 5′- CCACAGACCTTCCAGGAGAATG-3′,

IL-1β-Reverse: 5′- GTGCAGTTCAGTGATCGTACAGG-3′,

TNF-Forward: 5′- CTCTTCTGCCTGCTGCACTTTG-3′,

TNF-Reverse: 5′- ATGGGCTACAGGCTTGTCACTC-3′,

### Protein extraction and immune-blotting

Whole cell lysates were extracted with RIPA lysis buffer (P0013K, Beyotime) supplemented with protease and phosphatase inhibitor cocktail. Protein concentrations were determined by the Bradford assay. Soluble Protein (30–40 μg) was analyzed by SDS-PAGE followed by immunoblot analysis.

### Virus packaging and concentration

The lenti-virus plasmid (psPAX2 and pMD2.G) were co-transfected into HEK293T cells with VigoFect (Vigorous Biotechnology, Beijing, China). The cells were washed with fresh medium 6 h after transfection and cultured in fresh medium. The supernatant containing virus was collected after 24 h. All the retrovirus used in the mouse experiments were concentrated by centrifuging the viral medium at 27,500 rpm/min for 2 h, carefully removing the supernatant, and re-suspending the virus particles in a 100 μL of opti-mem medium. Shake gently at 4° C overnight.

### pGL3-P65-Luciferase reporter assay

500 ng of pGL3-P65-luciferase plasmid was mixed with 200 ng of renilla plasmid. The mixture was transfected into A549i cells seeded in 12-well plates via Lip3000. After 6 h, changed with fresh medium containing Dox for inducing ZNF24 expression for 12, 24 and 48 h. Cells were then digested with Trypsin and equal number of cells were then measured for luciferase activity.

### Chromatin immunoprecipitation (ChIP)-seq

The A549i cells were inoculated into eight 150-mm plates, and Dox was added to the final concentration of 1 μg/mL when the cell concentration reached 70–80%. After 2 days of culture, the cells were cross-linked with 1% formaldehyde (final concentration) and sonicated with the following parameters: 30 s on, 30 s off, 25% set power for 15 cycles. The total Lysis was divided into two bisected samples, one mixed with 1 mg/mL of Flag antibody and 40 μL of protein agarose bead. The other was mixed with 1 mg/mL of normal mice IgG (#F2416, Santa Cruz Biotechnology) and 40 μL of agarose beads. After overnight incubation, the beads were washed with a ChIP cleaning buffer. Chromatin was eluted, cross-linked DNA was reversed and DNA was extracted with phenol/chloroform. Finally, the eluted DNA was resolved in ddH2O and then sent to BGI (https://www.bgi.com/bgi-online) for sequencing.

### RNA sequencing

A549i cell was treated with or without 1 μg/mL of Dox for 48 h. The cells were digested with Trypsin. Total RNA was extracted with Trizol and sent to BGI for library construction and sequencing.

### Flow cytometry

To detect cell surface proteins (CD45, CD4, CD8, TIM-3), grind lung tissues separate from mice on grid 200. Collect cell suspension and use lysis solution (#NH4CL2009, TBD) to disrupt red blood cells. Then filter the cells to prepare a single cell suspension and staining antibody. For IL-2, IFNγ and TNF-α detection, firstly, stain the membrane proteins with antibodies against CD45, CD8 or CD4, wash once with PBS and use BD fixation and permeabilization kit (00–5523-00, eBioscience) overnight, then wash once with PBS, and stain with the corresponding antibody (IL-2, IFNγ, TNF-α). For CRT, HSP70 detection, EKVX-shZNF24 cells were treated with BAY11-7082, BI3406, or combination for 24 h. Cells were incubated with primary anti-CRT antibodies (1:100), or anti-HSP70 antibodies (1:100) for 1 h. The cells were washed and incubated with the FITC-conjugated monoclonal antibody for 30 min. Cells were analyzed by flow cytometry. For HMGB1 detection, EKVX-shZNF24 cells were treated with BAY11-7082, BI3406, or combination for 24 h. Cells were resuspended and fixed with the BD fixation and permeabilization kit (000-5523-00, eBioscience) overnight. These cells were then washed with PBS, stained with anti-HMGB1 antibodies for 1 h. The cells were washed and incubated with the FITC-conjugated monoclonal antibody for 30 min. Cells were analyzed by flow cytometry.

### CRISPR/Cas9 screen

We first infect EKVX cells with virus for encoding Cas9, and selected with blasticidin (3513–03-9, Solarbio) for two weeks. Monoclone was picked to generate a stable cell line (EKVX-Cas9). EKVX-Cas9 was further infected with the sgRNA library (addgene, #73178). After 24 h infection, a portion of the cells were collected as a control. The cells were selected with puromycin (1 μg/mL) for a week to eliminate uninfected cells. The infected cells were inoculated at 2 × 10^6^ cells subcutaneously into nude mice. After 14 days, the tumor was harvested to prepare genomic DNA. DNA fragment containing CRISPR sequence were PCR amplified and sent for sequencing.

### Histopathological analysis

Lung tissues were fixed with 10% neutral buffered formaldehyde (HT501320; Sigma-Aldrich) overnight, and dehydrated, embedded in paraffin. Then the tissues were sectioned for staining with hematoxylin and eosin (H&E) for histopathology.

### TCGA data analysis

We compared ZNF24 expression between normal tissue and primary tumor tissues was performed with UCSC Xena (http://xena.ucsc.edu/compare-tissue/). For correlation analysis between *ZNF24* and *RELA*, *RELB*, *CCNE1*, *CDKN1B*, *CDKN1A* in lung adenocarcinoma patients, we obtained the gene expression data from TCGA by UCSC Xena and the gene expression correlation was analyzed by GraphPad Prism 7.04. For the expression analysis of the *ZNF24* with *KRAS*, *EGFR* mutant in lung adenocarcinoma patients, patients were divided into high and low expression of ZNF24 according to the median expression of ZNF24.

### Survival curve analysis

The survival curve analysis of ZNF24 expression in lung cancer patients was usingKaplan-Meier-Plotter(https://kmplot.com/analysis/index.php?p=service&cancer=lung).

### Statistical analysis

Using GraphPad Prism 7.04 for statistics. Student’s t-test was used to compare differences between two experimental groups. Data are presented as means ± SEM and error bars denote SEM; n = 3; *P < 0.05; **P < 0.01; ***P < 0.001.

## Supplementary Information

**Additional file 1: Table S1.** CRISPR/Cas9 screen in EKVX-Cas9 cells for lung cancer tumor suppressor genes.

**Additional file 2: Table S2.** Clinical database from UCSC Xena(http://xena.ucsc.edu/) analysis of ZNF24 expression usingthe median value as a cutoff. Analysis the percentage patients with low expression of ZNF24 inpatients with KRAS mutations.

**Additional file 3: Table S3.** Clinical database from UCSC Xena(http://xena.ucsc.edu/) analysis ofZNF24 expression using the median value as a cutoff. Analysis thepercentage patients with low expression of ZNF24 inpatients with EGFR mutations.

**Additional file 4: Figure S1. ZNF24** is an essential tumor suppressor in lung cancer. **(A)** Western blot analysis of protein levels of Cas9 in EKVX cells. EKVX cells were infected with virus encoding Cas9, and selected with blasticidin for two weeks. Monoclone was picked to generate a stable cell line (EKVX-Cas9). **(B)** Scatter diagram showing genes targeted by sgRNAs that were differentially depleted or enriched in EKVX-Cas9 cells. **(C)** KEGG analysis of the top enriched 230 genes in genome-wide screening through CRISPR/Cas9. **(D)** RT-qPCR detect the knock-down efficiency of *ZNF24*, *NR3C2*, *CST4*, *ARHGDIG* and *CRYBB3*. RNA was extracted from engineered EKVX cells. Expression of the *ZNF24*, *NR3C2*, *CST4*, *ARHGDIG* and *CRYBB3* were quantified through RT-qPCR. **(E-I)** Impact of expression level of ZNF24, NR3C2, CST4, ARHGDIG and CRYBB3 on proliferation of EKVX cells. Engineered EKVX cells were seeded in 96-well plates and cultured for 4 days. Cell viability was analyzed with CCK8. shGFP as control knockdown. **(J)** Comparison of protein levels of ZNF24 in tumors, para-tumoral lung tissues and lung cancer cell lines. Expression level of ZNF24 was checked through western blotting analysis on tumors, para-tumoral lung tissues (upper panel). Evaluation of ZNF24 expression with RT-qPCR in various lung cancer cell lines (lower panel). RNA was extracted from various lung cancer cell lines. Expression of the ZNF24 were quantified through RT-qPCR. Bars are represented as mean ± SEM of the indicated number (n) of repeats. *P<0.05, **P<0.01, and ***P<0.001 by Student’s t-test. **Figure S2.** *ZNF24* is an essential tumor suppressor in lung cancer. **(A)** Western Blot evaluation of ZNF24 expression induced by Dox (1 μg/mL) for 48 h in A549i and Hop62i cells. **(B)** Impact of ZNF24 expression level on proliferation of A549i and Hop62i cells. A549, Hop62 infected with lenti-virus for expressing ZNF24 (designated A549i, Hop62i). A549i, Hop62i (1000 cells) were respectively inoculated in 96-well plates and cultured with 1 μg/mL of Dox for 5 days. Cell viability was analyzed with CCK8. **(C)** Impact of ZNF24 expression level on colony forming ability of Hop62i cells. Hop62i (1000 cells) were seeded in 6-well plates and treated with Dox (1 μg/mL) for 2 weeks before quantification for colonies. Left: representative pictures. Right: statistics of colony number. **(D)** Validation of knockdown efficiency of three ZNF24 shRNAs. 293T cells were co-transfected with pCDNA3.1-ZNF24 and one of three plasmids encoding shZNF24s. Expression level of ZNF24 was analyzed through Western Blot. shRNA A exhibited the highest knockdown efficiency. **(E)** Western blot detecting the knockdown and replenishing efficiency of ZNF24 in EKVX cells. **(F)** Effect of ZNF24 knockdown or re-expression on proliferation of H322 cells. 1000 engineered H322 cells were seeded in 96-well plates and cultured for 5 days. Cell viability was analyzed with CCK8. shGFP as control knockdown; shZNF24 for ZNF24 knockdown; sh/+ZNF24 for ZNF24 re-expression in ZNF24 knockdown cells. **(G)** Impact of ZNF24 knockdown or re-expression on 2-D colony formation ability of H322 cells. 1000 engineered H322 cells were respectively inoculated in 6-well plates and cultured for 2 weeks before quantification for colonies. Left: representative pictures. Right: statistics of colony number. **(H)** Nontoxic effect of Dox on the weight of nude mice. **(I)** Impact of ZNF24 on EKVX xenografted tumors. Cells (2×10^6^) were inoculated into nude mice for tumor growth for 14 days. Tumor volume is recorded every 2 days. A representative image of the tumors at the end of the experiments is shown. **(J-K)** The tumor growth or weight were monitored of **(I)**. **(L)** Expression of ZNF24 in lungs of KC-C; KC-Z transgenic mice revealed through IHC staining (left) and western blot (right). **(M)** Knockout efficiency of ZNF24 by lenti-virus encoded CRISPR confirmed through Sanger sequencing. **(N)** IHC staining of ZNF24 in mouse section of K-sgTD mice and K-sgZNF24 mice (left). Western blot detecting the knockout efficiency of ZNF24 in K-sgTD mice and K-sgZNF24 mice (right). Bars are represented as mean ± SEM of the indicated number (n) of repeats. *P<0.05, **P<0.01, and ***P<0.001 by Student’s t-test. **Figure S3.** Ectopic expression of ZNF24 induces cell-cycle arrest of lung cancer cell. **(A)** Impact of ZNF24 expression on cell apoptosis in A549i, Hop62i cells. A549i, Hop62i cells were treated with Dox (1 μg/mL) for 48 h. Flow cytometry detection of the effect of overexpression of ZNF24 on cell apoptosis. **(B)** Western blot evaluation of expression of apoptosis-related proteins. A549i, Hop62i cells were treated with Dox (1 μg/mL) for 48 h. The cells were harvested for immunoblot analysis with indicated antibodies. **(C)** Impact of ZNF24 expression on cell pyroptosis in A549i, Hop62i cells. A549i, Hop62i cells were treated with Dox (1 μg/mL) for 48 h. The cells were harvested for immunoblot analysis with indicated antibodies. **(D)** Impact of ZNF24 expression on cell necroptosis in A549i, Hop62i cells. A549i, Hop62i cells were treated with Dox (1 μg/mL) for 48 h. The cells were harvested for immunoblot analysis with indicated antibodies. **(E)** Impact of ZNF24 expression on senescence in A549i and Hop62i cells. A549i and Hop62i cells were treated with Dox (1 μg/mL) for 48 h before β-galactosidase staining. Representative pictures (left) and statistics (right). **(F)** Impact of ZNF24 expression on DNA damage in A549i, Hop62i cells. A549i and Hop62i cells were treated with Dox (1 μg/mL) for 48 h. Western Blot detected γ-H2AX in A549i and Hop62i cells. **(G)** Impact of ZNF24 expression on proliferation of EKVX and H322 cells. Cells were cultured in media containing 10 μM of Edu dye. Cell proliferation was evaluated by checking the incorporation of Edu through fluorescent microscopy. **(H)** Statistics of **(G)**. **(I)** RT-qPCR analysis of impact of ZNF24 expression on* CDK2*, *Cyclin D1* and *Cyclin E1* expression in EKVX, H322 cells. Bars are represented as mean ± SEM of the indicated number (n) of repeats. *P<0.05, **P<0.01, and ***P<0.001 by Student’s t-test. **Figure S4.** ZNF24 induces cell cycle arrest through NF-κB signaling pathways. **(A)** The volcano map representation of impact of ZNF24 expression on transcriptome of A549i cells. A549i cells were treated with 1 μg /mL of Dox for 48 h. RNAs were isolated and subjected to RNA-sequencing. **(B)** Impact of ZNF24 expression on NF-κB reporter in Hop62i cells. Hop62i cells were transfected with the NF-κB reporter. Luciferase assays were performed 12, 24 and 48 h after inducing with Dox (1 μg/mL). **(C)** Knock-down of ZNF24 in H322 cells sensitized to the inhibitors of NF-κB. H322-shGFP, H322-shZNF24, H322-sh/+ZNF24 cells (1000) were seeded in six-well plates and treated with DMSO or NF-κB inhibitor (BAY11-7082, 2 μM) for 2 weeks. Cells were stained with 0.5% crystal violet. **(D)** Statistics of **(C)**. **(E**-**F)** Impact of NF-κB inhibitor on expression of cycle-related proteins by lung cancer cells. RT-qPCR detection of expression on cycle related genes. After treatment of EKVX-shZNF24 and H322-shZNF24 cells with BAY11-7082 (2 μM) for 24 h. E. data on EKVX-shZNF24 cells. F. data on H322-shZNF24 cells. **(G-H)** Impact of NF-κB inhibitor on transcription of target genes (*IL-1β*, *IL-6*, *TNFα*) by EKVX-shZNF24 and H322-shZNF24 cells. RT-qPCR analysis of expression of related genes after treatment of EKVX-shZNF24 and H322-shZNF24 cells with BAY11-7082 (2 μM) for 24 h. G. data on EKVX-shZNF24 cells. H. data on H322-shZNF24 cells. Bars are represented as mean ± SEM of the indicated number (n) of repeats. *P<0.05, **P<0.01, and ***P<0.001 by Student’s t-test. **Figure S5**. ZNF24 binds *P65* promoter to negatively regulate its expression. **(A-B)** Genome-wide analysis of ZNF24 binding sites in lung cancer cells through ChIP-seq analysis. A549i cells were treated with Dox (1 μg/mL) for 48 h. ChIP-seq was performed on DNA samples enriched for the Flag antibody. ZNF24 bound the promoter region of *VEGFA* **(A)** and *PDGFRB* **(B)** genes. **(C)** Impact of ZNF24 expression on activity of *P65*.* P65* promoter region was cloned into pGL3-Luciferase plasmid (designated pGL3-*P65*-Luciferase). The construct (0.5 μg) was transfected into A549 cells together with ZNF24 expression plasmid (0.5 mg). Luciferase activity was monitored 12, 24 and 48 h later. **(D)** Impact of P65 expression of cell cycle in Hop62i and Hop62i-P65 cells. Hop62i and Hop62i-P65 cells treated with Dox (1 μg/mL) for 48 h. Cell cycle were determined through FACS analysis of DNA contents revealed by propidium Iodide (PI) staining. Results are represented as percent of cell population in G0/G1, S and G2/M phases of the cell cycle. **(E)** Statistics of **(D)**. **(F-G)** Impact of P65 expression on cyclin-associated genes and NF-κB target genes in A549i-P65 and Hop62i-P65 cells. A549i-P65 and Hop62i-P65 cells were treated with Dox (1 μg/mL) for 48 h. RNA was extracted from cells. Expression of the indicated genes were quantified through RT-qPCR. F. data on A549i cells. G. data on Hop62i cells. Bars are represented as mean ± SEM of the indicated number (n) of repeats. *P<0.05, **P<0.01, and ***P<0.001 by Student’s t-test. **Figure S6.** Combinational inhibition of KRAS, NF-κB and PD-1 effectively shrinks Kras^G12D^/ZNF24^-/-^ lung cancers. **(A-C)** Impact of the growth of EKVX-shZNF24 cells in nude mice treated BAY11-7082 synergizes with BI3406. EKVX-shZNF24 cells 2×10^6^ were inoculated into nude mice. Mice were treated with BAY11-7082 (20 mg/kg/day, intraperitoneal injection), BI3406 (25 mg/kg/day, gavage), and combination for 2 weeks. The xenografts were dissected to images **(A)** and the tumor growth or weight were monitored **(B-C)**. **(D)** Combination of BAY11-7082 and BI3406 is limited efficacy to shrink lung tumor in K-sgZNF24 mice. The recombinant lenti-virus co-expressed Cre and CRISPR/Cas9 to infect Lsl-Kras^G12D^ through nasal inhalation. Tumor burdens were documented with CT. BAY11-7082 (20 mg/kg/day, intraperitoneal injection), BI3406 (25 mg/kg/day, gavage) were administered. **(E)** Quantification of relative tumor burden of mice of **(D)**. **(F)** Representative images of Hematoxylin and eosin (H&E) staining of the lung tissue obtained from different treatment groups. **(G-H)** Quantification of tumor numbers and repression of tumor size of K-sgZNF24 mice lung cancers. **(I-K)** Impact of the growth of LLC-shZNF24 cells in nude mice treated BAY11-7082 synergizes with BI3406. LLC-shZNF24 cells 2×10^6^ were inoculated into nude mice. Mice were treated with BAY11-7082 (20 mg/kg/day, intraperitoneal injection), BI3406 (25 mg/kg/day, gavage), and combination for 2 weeks. The xenografts were dissected to images **(I)** and the tumor growth or weight were monitored (**J-K)**. **(L-N)** Impact of the growth of LLC-shZNF24 cells in wild-type mice treated BAY11-7082 synergizes with BI3406. LLC-shZNF24 cells 2×10^6^ were inoculated into wild-type mice. Mice were treated with BAY11-7082 (20 mg/kg/day, intraperitoneal injection), BI3406 (25 mg/kg/day, gavage), and combination for 2 weeks. The xenografts were dissected to images **(L)** and the tumor growth or weight were monitored **(M-N)**. Figure S7. Combinational inhibition of KRAS, NF-κB and PD-1 effectively shrinks Kras^G12D^/ZNF24^-/-^ lung cancers. **(A-C) **Impact of the growth of LLC-shZNF24/shP65 cells in wild-type mice treated BAY11-7082 synergizes with BI3406. LLC-shZNF24/shP65 cells 2×10^6^ were inoculated into wild-type mice. Mice were treated with BAY11-7082 (20 mg/kg/day, intraperitoneal injection), BI3406 (25 mg/kg/day, gavage), and combination for 2 weeks. The xenografts were dissected to images **(A)** and the tumor growth or weight were monitored **(B-C)**. **(D)** RT-qPCR analysis of impact of BAY11-7082 on expression of NF-κB target genes. A549 cells were treated with BAY11-7082 for 24h.* IL-1β*, *IL-6*,* TNFα* expression was quantified through RT-qPCR analysis. **(E)** Impact of PD-L1 expression on BAY11-7082 treatment in A549 cells. A549 cells were treated with BAY11-7082 for 24h. Expression of PD-L1 was detected by flow cytometry. **(F)** Induction of immunogenic cell death by NF-κB inhibitor (BAY11-7082) and/ or KRAS inhibitor (BI3406) in EKVX-shZNF24 cells. EKVX-shZNF24 cells were treated with BAY (2 μM) and/or BI3406 (1 μM) for 24 h. Expression of HMGB1 was determined by flow cytometry. **(G**) Induction of immunogenic cell death by NF-κB inhibitor (BAY11-7082) and/ or KRAS inhibitor (BI3406) in EKVX-shZNF24 cells. Levels of extracellular ATP in treated EKVX-shZNF24 cells measured by luminescence. **(H)** Anti-mouse PD-1 antibody alone is limited efficacy to shrink lung tumor in K-sgZNF24 mice. The recombinant lenti-virus co-expressed Cre and CRISPR/Cas9 to infect Lsl-Kras^G12D^ through nasal inhalation. Tumor burdens were documented with CT. Anti-mouse PD-1 (5 mg/kg, every other day, intraperitoneal) were administered. **(I) **Quantification of relative tumor burden of mice of **(H)**. **(J)** Western blot detecting the knockout efficiency of ZNF24 in K-sgZNF24 mice indicated treated. **Figure S8.** Combinational inhibition of KRAS, NF-κB and PD-1 effectively shrinks Kras^G12D^/ZNF24^-/-^ lung cancers. **(A-B)** Impact of infiltration of CD4+ T cells and CD8+ T cells in K-sgTD and K-sgZNF24 mice. **(C)** Deletion of CD4+ T cells by CD4 antibody in K-sgZNF24. CD4+ T cells in peripheral blood were detected after 2 weeks of treatment. **(D)** Deletion of CD8+ T cells by CD8 antibody in K-sgZNF24. CD8+ T cells in peripheral blood were detected after 2 weeks of treatment. **(E)** Impact of CD4 T cells and CD8 T cells on combinational treatment with BAY11-7082, BI3406 and PD-1 antibody in K-sgZNF24 mice. K-sgZNF24 mice were treated with combination (BAY11-7082, BI3406 and PD-1 antibody) or combinational plus CD4/8 antibody. **(F)** Quantification of tumor burden of mice of Combination of **(E)**. **(G) **Gating strategy for analyzing CD4 and CD8 positive T cells in lung tissues. **(H)** Impact of the combination of BAY11-7082, BI3406, anti-PD-1 (designated BAY+BI+PD-1) on infiltration of CD8+ T cells in lung cancer. Lung tissue were dissected for analyze the infiltration of CD8+ T cells in tumors by flow cytometry. **(I)** Impact of the combination of BAY11-7082, BI3406, anti-PD-1 (designated BAY+BI+PD-1) on infiltration of CD4+ T cells in lung cancer. Lung tissue were dissected for analyze the infiltration of CD4+ T cells in tumors by flow cytometry. **(J-K)** Combinational treatment with BAY11-7082, BI3406 and PD-1 antibody (designated BAY+BI+PD-1) activated expression of effector cytokine in tumor infiltrating CD8+ T cells. Tumor-infiltrating CD8+ T cells were intracellularly stained for FACS analysis of expression of IFNγ and TNF-α. Bars are represented as mean ± SEM of the indicated number (n) of repeats. *P<0.05, **P<0.01, and ***P<0.001 by Student’s t-test. **Figure S9.** ZNF24-NF-κB signaling axis is clinically relevant. **(A)** Expression of human ZNF24, P65, PD-1, PD-L1 in lung cancer patients revealed through IHC staining. **(B)** Correlation between expression of ZNF24 and P65. Expression data of lung cancer patients from IHC staining by Average Optical Density (AOD) with Image J. **(C)** Correlation between expression of PD-L1 and ZNF24. Expression data of lung cancer patients from IHC staining by Average Optical Density (AOD) with Image J. **(D)** Correlation between expression of PD-L1 and P65. Expression data of lung cancer patients from IHC staining by Average Optical Density (AOD) with Image J.

## Data Availability

All the data obtained and/or analyzed during the current study were available from the corresponding authors on reasonable request.

## Abbreviations

SCLC: Small cell lung cancer
NSCLC: Non‐small cell lung cancer
TFs: Transcription factors
ZNF24: Zinc finger protein 24
TSGs: Tumor suppressor genes
KEGG: Kyoto encyclopedia of genes and genomes
CFSE: Carboxy fluoroscein succinimidyl ester
RT-qPCR: Reverse transcription-quantitative PCR
RNA-seq: RNA sequencing
ChIP-seq: Chromatin immunoprecipitation sequencing
ICD: Immunogenic cell death
CRT: Calreticulin
CT: Computed tomography
FACS: Fluorescence-activated single cell sorting
IFNγ: Interferon gamma
NF-κB: Nuclear factor kappa-light-chain-enhancer of activated B cells
PD1: Programmed death-1
KRAS: Kirsten rat sarcoma virus
EGFR: Epidermal growth factor receptor
LUAD: Lung adenocarcinoma
TCGA: The cancer genome atlas
DCs: Dendritic cells
IHC: Immunohistochemistry
H&E: Hematoxylin and eosin
shRNA: Short hairpin RNA
sgRNA: Single guide RNA
GAPDH: Glyceraldehyde-3-phosphate dehydrogenase
