# Supplementary material for: In vivo genome-wide CRISPR screening identifies ZNF24 as a negative NF-κB modulator in lung cancer
Source: Cell Biosci. 2022 Dec 1;12:193. doi: 10.1186/s13578-022-00933-0 (PMC9717477; doi:10.1186/s13578-022-00933-0)
Supplement: Supplementary file 1 — Additional file 1: Table S1. CRISPR/Cas9 screen in EKVX-Cas9 cells for lung cancer tumor suppressor genes. [file 13578_2022_933_MOESM1_ESM.pdf]

TableS1 CRISPR/Cas9 screen in EKVX-Cas9 cells for lung cancer tumor suppressor genes

GeneSymbol	fold change(fc)
ZNF24_20525	16.19577639
NR3C2_11576	10.92816901
CST4_4135	10.89466292
ARHGDIG_1149	10.84834835
CRYBB3_3967	10.70833333
ITGB5_9817	10.62534819
CPTP_51340	9.964169381
SOX3_17979	8.857819905
RNF223_72518	8.66252588
FFAR3_7625	8.053571429
GSK3B_7853	8
KIAA0040_25571	7.979567308
DMRTC1_47209	6.681415929
CDH11_2870	6.565552699
ASL_1260	6.466019417
ROBO2_16235	6.182706767
LRP1_10785	6.129032258
MTSS1L_56131	6.05853358
KLF6_3681	6.012345679
SOAT1_17929	5.997150997
CLUAP1_31604	5.895280236
NR0B1_535	5.854368932
GLYR1_53948	5.722560976
M6PR_10925	5.463636364
NARFL_47785	5.427607362
C1QL2_63829	5.423143351
TXNDC16_45794	5.314327485
GPN2_40421	5.276397516
RRM2_16650	5.235546039
TFB2M_47538	5.206939281
TRAP1_27106	5.171003717
HNRNPC_8585	5.131889764
PPT1_14695	5.067901235
INPP5E_44299	5.022550544
PTPRC_15466	5.01981982
MOGAT1_57714	5.007194245
C5orf28_47744	4.97029703
SMIM7_48900	4.964238411
TBC1D10C_70280	4.947867299
CDC20_2801	4.890034364
MS4A10_69286	4.869565217
FAM172A_52750	4.829032258
CAV1_2386	4.813043478
SHC1_17307	4.792349727
S100A1_16710	4.778597786
STIM1_18396	4.70292887
DUSP4_5155	4.701910828
FIGLA_69582	4.688227684
LYSMD2_66787	4.687804878
TCOF1_18892	4.685224839
RFPL4B_73253	4.667660209
ADH7_360	4.657320872

GSX2_64232	4.591911765
DUSP2_5147	4.542628774
ZNF558_61962	4.534456355
TRIP4_24491	4.527131783
HBA2_8192	4.471686747
TGIF2LY_55579	4.457786116
MFRP_52238	4.438735178
C2orf71_71220	4.428346457
OR7A10_71871	4.419182948
COPB2_24378	4.410958904
OR2T1_35267	4.393869732
HOXB13_28234	4.390322581
LTBR_10854	4.390070922
ALOX5AP_671	4.348378894
KCNK7_26968	4.328482328
MSANTD3_55780	4.280487805
CSE1L_4007	4.278026906
ADNP_32677	4.271226415
SDHAF1_73900	4.23345367
SFMBT1_38813	4.133504493
IFNA1_9201	4.117647059
AAGAB_49737	4.116550117
CCDC186_41530	4.070941337
MPST_11723	4.049450549
HRSP12_27508	4.047003525
GOLGB1_7425	4.011164274
PRPF6_33631	3.970703125
ILVBL_29792	3.939271255
KRT72_60985	3.921126761
IPO11_38101	3.916326531
ALS2CL_66929	3.905511811
ADAM15_22888	3.878318584
PIKFYVE_64739	3.865187713
CLYBL_64287	3.864321608
C9orf3_54446	3.863484087
CH25H_23616	3.794964029
CDH2_2834	3.793696275
ADGRE3_53951	3.78685259
HDAC5_26733	3.75896861
MAGEA3_11006	3.671428571
CHST4_27221	3.654320988
TM6SF1_39556	3.649717514
AJAP1_43788	3.606818182
AADAC_23	3.598958333
ELF5_5517	3.585213033
WDR7_32497	3.583255814
STAP1_34785	3.580547112
CTSC_3076	3.571428571
CMPK1_39381	3.5625
CRB3_56232	3.546
TYRO3_19793	3.544715447
KLHL24_40600	3.541528239
RPS10-NUDT3_76115	3.534090909
SACS_34927	3.49197861

UQCRH_20036	3.482889734
AP1B1_460	3.449275362
RANBP1_15790	3.443181818
FOXP4_57644	3.396638655
RFX7_48212	3.372168285
RTEL1_39424	3.321212121
NUDT11_41866	3.315463918
JMJD8_68851	3.305732484
DSTN_29911	3.270928463
SPC25_45419	3.162454874
TTC1_19679	3.135881104
TTC7A_45304	3.1
MAZ_11170	3.061876248
ATP13A5_69655	3.04137931
ZSWIM8_31593	3.024752475
LOC400736_72259	3.004893964
PDYN_13639	3
PPRC1_31678	3
TNNT2_19412	3
SERPINA5_13414	2.979253112
PCYOX1_38787	2.96124031
BMP3_1778	2.949880668
FAM134A_48978	2.935779817
C7orf25_48728	2.934497817
NKX6-3_63070	2.919811321
KLK3_1003	2.915909091
AMFR_728	2.91521197
LRRC74A_61436	2.908831909
C4A_1985	2.902654867
LHCGR_10625	2.890466531
DNAH7_44100	2.870748299
DAP3_20960	2.850574713
NOL7_38706	2.83
OR6S1_69349	2.82967033
CHMP3_39230	2.828877005
SLC31A1_3685	2.823076923
WDR76_50487	2.817120623
AMY1B_765	2.802564103
BTBD10_53328	2.797927461
KLK8_30470	2.778251599
EIF4A1_5436	2.742424242
TIMM21_36441	2.736220472
ASCL5_74189	2.728110599
XRCC4_20373	2.712560386
ZNF492_46027	2.695652174
CROT_40397	2.69375
GFRA1_7076	2.672985782
SHMT1_17319	2.664031621
PSMC2_15218	2.637614679
MAGEA2_11002	2.635933806
SH3BP4_33461	2.572938689
SERPINB8_13912	2.563492063
PARD3_44214	2.53125
CCDC167_62898	2.522727273

OR3A2_13091	2.450381679
TMEM223_48832	2.443983402
TNRC18_53881	2.420310296
XBP1_20327	2.409090909
WARS2_27837	2.389830508
KANSL1L_62429	2.333333333
IER5_38334	2.326203209
DHX40_49564	2.316455696
GDE1_39066	2.302083333
NUPL2_30110	2.29305136
ARMCX5-GPRASP2_76092	2.262411348
NUP160_32324	2.221238938
PTGR2_61426	2.207207207
TMEM161A_41027	2.18061674
FABP2_6007	2.176870748
IL17RD_40501	2.151724138
ZNF784_61872	2.137195122
NDN_12257	2.118794326
RDH12_61392	2.097701149
TM4SF1_10920	2.050377834
CASC1_42115	2.048913043
GABRG2_6787	2.04494382
ZNF676_63664	2.035714286
BOLA1_37583	2.01396648
AICDA_45389	2
BMP1_1772	2
ZNF343_49081	2
KLF5_1913	1.991304348
ST18_25683	1.949367089
MED18_40589	1.94047619
RARA_15831	1.932291667
PDCL3_48760	1.913690476
MISP_59127	1.885714286
ZNF24_20526	1.882723784
SLC36A2_62752	1.866348449
GSTM5_7905	1.857142857
CECR6_36139	1.856502242
MICB_11483	1.835341365
NUDT4_30330	1.827586207
HYPM_33654	1.818348624
GAB1_6722	1.783783784
PSPH_15296	1.77540107
FAM45A_72684	1.751937984
MBTPS1_22779	1.744680851
C11orf16_44369	1.734463277
TRIM37_11931	1.707482993
PTGER1_15328	1.695652174
LRFN1_46054	1.689373297
CCDC7_49812	1.674858223
GPR39_7618	1.663366337
DDIT3_4623	1.630630631
C14orf105_41883	1.616497829
CRHBP_3886	1.602564103
PDX1_9675	1.587962963

NME9_69881	1.570652174
TCERG1L_66783	1.551470588
IFNA10_9230	1.551020408
CCR1_3442	1.549668874
CRIP1_3899	1.535714286
GJA5_7153	1.531034483
STAT6_18367	1.529255319
FOXR1_67223	1.529220779
ASB9_60813	1.508928571
CKB_3279	1.504273504
ARAF_1059	1.5
EVA1B_41881	1.5
GLRX3_28423	1.5
WARS_20212	1.5
CDIPT_28035	1.5
PUS7_40134	1.5
