# Supplementary material for: In vivo genome-wide CRISPR screening identifies ZNF24 as a negative NF-κB modulator in lung cancer
Source: Cell Biosci. 2022 Dec 1;12:193. doi: 10.1186/s13578-022-00933-0 (PMC9717477; doi:10.1186/s13578-022-00933-0)
Supplement: Supplementary file 2 — Additional file 2: Table S2. Clinical database from UCSC Xena(http://xena.ucsc.edu/) analysis of ZNF24 expression usingthe median value as a cutoff. Analysis the percentage patients with low expression of ZNF24 inpatients with KRAS mutations. [file 13578_2022_933_MOESM2_ESM.pdf]

Table S2 Clinical database from UCSC Xena(http://xena.ucsc.edu/) analysis of ZNF24 expression using the median value as a cutoff. Analysis the percentage patients with low expression of ZNF24 in patients with KRAS mutations.

samples	_study	_sample_type	_primary_ site	primary disease or tissue	ZNF24expression	KRAS mutation
TCGA-86-7713-01	TCGA	Primary Tumor	Lung	Lung Adenocarcinon	15.05	KRAS
TCGA-55-1595-01	TCGA	Primary Tumor	Lung	Lung Adenocarcinon	14.93	KRAS
TCGA-MN-A4N5-01	TCGA	Primary Tumor	Lung	Lung Adenocarcinon	14.75	KRAS
TCGA-95-7567-01	TCGA	Primary Tumor	Lung	Lung Adenocarcinon	14.74	KRAS
TCGA-99-8033-01	TCGA	Primary Tumor	Lung	Lung Adenocarcinon	13.59	KRAS
TCGA-44-3917-01	TCGA	Primary Tumor	Lung	Lung Adenocarcinon	13.56	KRAS
TCGA-67-3773-01	TCGA	Primary Tumor	Lung	Lung Adenocarcinon	13.36	KRAS
TCGA-97-7938-01	TCGA	Primary Tumor	Lung	Lung Adenocarcinon	13.34	KRAS
TCGA-69-7980-01	TCGA	Primary Tumor	Lung	Lung Adenocarcinon	13.25	KRAS
TCGA-97-7554-01	TCGA	Primary Tumor	Lung	Lung Adenocarcinon	13.24	KRAS
TCGA-78-7540-01	TCGA	Primary Tumor	Lung	Lung Adenocarcinon	13.12	KRAS
TCGA-55-A494-01	TCGA	Primary Tumor	Lung	Lung Adenocarcinon	13.09	KRAS
TCGA-05-4390-01	TCGA	Primary Tumor	Lung	Lung Adenocarcinon	13.05	KRAS
TCGA-53-7813-01	TCGA	Primary Tumor	Lung	Lung Adenocarcinon	13.05	KRAS
TCGA-55-7815-01	TCGA	Primary Tumor	Lung	Lung Adenocarcinon	13.03	KRAS
TCGA-55-7914-01	TCGA	Primary Tumor	Lung	Lung Adenocarcinon	13.02	KRAS
TCGA-55-8097-01	TCGA	Primary Tumor	Lung	Lung Adenocarcinon	13.02	KRAS
TCGA-55-6970-01	TCGA	Primary Tumor	Lung	Lung Adenocarcinon	13.01	KRAS
TCGA-62-A46S-01	TCGA	Primary Tumor	Lung	Lung Adenocarcinon	12.95	KRAS
TCGA-75-6206-01	TCGA	Primary Tumor	Lung	Lung Adenocarcinon	12.88	KRAS
TCGA-J2-A4AG-01	TCGA	Primary Tumor	Lung	Lung Adenocarcinon	12.83	KRAS
TCGA-05-4244-01	TCGA	Primary Tumor	Lung	Lung Adenocarcinon	12.82	KRAS
TCGA-44-6146-01	TCGA	Primary Tumor	Lung	Lung Adenocarcinon	12.81	KRAS
TCGA-55-7911-01	TCGA	Primary Tumor	Lung	Lung Adenocarcinon	12.78	KRAS
TCGA-55-6984-01	TCGA	Primary Tumor	Lung	Lung Adenocarcinon	12.75	KRAS
TCGA-91-6828-01	TCGA	Primary Tumor	Lung	Lung Adenocarcinon	12.73	KRAS
TCGA-44-8117-01	TCGA	Primary Tumor	Lung	Lung Adenocarcinon	12.73	KRAS
TCGA-69-7973-01	TCGA	Primary Tumor	Lung	Lung Adenocarcinon	12.7	KRAS
TCGA-86-8054-01	TCGA	Primary Tumor	Lung	Lung Adenocarcinon	12.7	KRAS
TCGA-64-5774-01	TCGA	Primary Tumor	Lung	Lung Adenocarcinon	12.66	KRAS
TCGA-69-8254-01	TCGA	Primary Tumor	Lung	Lung Adenocarcinon	12.66	KRAS
TCGA-95-A4VK-01	TCGA	Primary Tumor	Lung	Lung Adenocarcinon	12.65	KRAS
TCGA-NJ-A4YI-01	TCGA	Primary Tumor	Lung	Lung Adenocarcinon	12.6	KRAS
TCGA-78-7148-01	TCGA	Primary Tumor	Lung	Lung Adenocarcinon	12.58	KRAS
TCGA-55-7281-01	TCGA	Primary Tumor	Lung	Lung Adenocarcinon	12.52	KRAS
TCGA-95-A4VP-01	TCGA	Primary Tumor	Lung	Lung Adenocarcinon	12.52	KRAS
TCGA-97-A4M5-01	TCGA	Primary Tumor	Lung	Lung Adenocarcinon	12.5	KRAS
TCGA-93-A4JO-01	TCGA	Primary Tumor	Lung	Lung Adenocarcinon	12.5	KRAS
TCGA-73-4662-01	TCGA	Primary Tumor	Lung	Lung Adenocarcinon	12.49	KRAS
TCGA-43-2581-01	TCGA	Primary Tumor	Lung	Lung Squamous Cell	12.49	KRAS
TCGA-L9-A443-01	TCGA	Primary Tumor	Lung	Lung Adenocarcinon	12.46	KRAS
TCGA-93-7347-01	TCGA	Primary Tumor	Lung	Lung Adenocarcinon	12.45	KRAS
TCGA-MP-A4TF-01	TCGA	Primary Tumor	Lung	Lung Adenocarcinon	12.45	KRAS
TCGA-55-8514-01	TCGA	Primary Tumor	Lung	Lung Adenocarcinon	12.45	KRAS
TCGA-55-8508-01	TCGA	Primary Tumor	Lung	Lung Adenocarcinon	12.44	KRAS
TCGA-67-3774-01	TCGA	Primary Tumor	Lung	Lung Adenocarcinon	12.44	KRAS
TCGA-55-7284-01	TCGA	Primary Tumor	Lung	Lung Adenocarcinon	12.44	KRAS
TCGA-86-A456-01	TCGA	Primary Tumor	Lung	Lung Adenocarcinon	12.44	KRAS
TCGA-64-5815-01	TCGA	Primary Tumor	Lung	Lung Adenocarcinon	12.42	KRAS

TCGA-NJ-A4YG-01	TCGA	Primary Tumor	Lung	Lung Adenocarcinon	12.41	KRAS	
TCGA-99-8025-01	TCGA	Primary Tumor	Lung	Lung Adenocarcinon	12.4	KRAS	
TCGA-86-A4JF-01	TCGA	Primary Tumor	Lung	Lung Adenocarcinon	12.37	KRAS	
TCGA-95-A4VN-01	TCGA	Primary Tumor	Lung	Lung Adenocarcinon	12.36	KRAS	
TCGA-55-8615-01	TCGA	Primary Tumor	Lung	Lung Adenocarcinon	12.35	KRAS	
TCGA-55-6642-01	TCGA	Primary Tumor	Lung	Lung Adenocarcinon	12.34	KRAS	
TCGA-55-7725-01	TCGA	Primary Tumor	Lung	Lung Adenocarcinon	12.34	KRAS	$ZNF24^{high}$
TCGA-80-5608-01	TCGA	Primary Tumor	Lung	Lung Adenocarcinon	12.34	KRAS	
TCGA-93-8067-01	TCGA	Primary Tumor	Lung	Lung Adenocarcinon	12.34	KRAS	
TCGA-66-2769-01	TCGA	Primary Tumor	Lung	Lung Squamous Cell	12.34	KRAS	
TCGA-55-7910-01	TCGA	Primary Tumor	Lung	Lung Adenocarcinon	12.33	KRAS	
TCGA-35-3615-01	TCGA	Primary Tumor	Lung	Lung Adenocarcinon	12.31	KRAS	
TCGA-69-7978-01	TCGA	Primary Tumor	Lung	Lung Adenocarcinon	12.31	KRAS	
TCGA-86-7953-01	TCGA	Primary Tumor	Lung	Lung Adenocarcinon	12.31	KRAS	
TCGA-44-7661-01	TCGA	Primary Tumor	Lung	Lung Adenocarcinon	12.29	KRAS	
TCGA-86-8359-01	TCGA	Primary Tumor	Lung	Lung Adenocarcinon	12.29	KRAS	
TCGA-MP-A4SY-01	TCGA	Primary Tumor	Lung	Lung Adenocarcinon	12.26	KRAS	
TCGA-69-8253-01	TCGA	Primary Tumor	Lung	Lung Adenocarcinon	12.25	KRAS	
TCGA-55-8207-01	TCGA	Primary Tumor	Lung	Lung Adenocarcinon	12.24	KRAS	
TCGA-4B-A93V-01	TCGA	Primary Tumor	Lung	Lung Adenocarcinon	12.23	KRAS	
TCGA-75-7030-01	TCGA	Primary Tumor	Lung	Lung Adenocarcinon	12.23	KRAS	
TCGA-69-7974-01	TCGA	Primary Tumor	Lung	Lung Adenocarcinon	12.23	KRAS	
TCGA-55-6983-01	TCGA	Primary Tumor	Lung	Lung Adenocarcinon	12.22	KRAS	
TCGA-55-8299-01	TCGA	Primary Tumor	Lung	Lung Adenocarcinon	12.22	KRAS	
TCGA-78-7539-01	TCGA	Primary Tumor	Lung	Lung Adenocarcinon	12.22	KRAS	
TCGA-NJ-A55R-01	TCGA	Primary Tumor	Lung	Lung Adenocarcinon	12.22	KRAS	
TCGA-55-8512-01	TCGA	Primary Tumor	Lung	Lung Adenocarcinon	12.21	KRAS	
TCGA-73-4677-01	TCGA	Primary Tumor	Lung	Lung Adenocarcinon	12.21	KRAS	
TCGA-49-4505-01	TCGA	Primary Tumor	Lung	Lung Adenocarcinon	12.19	KRAS	
TCGA-50-5941-01	TCGA	Primary Tumor	Lung	Lung Adenocarcinon	12.18	KRAS	
TCGA-55-8090-01	TCGA	Primary Tumor	Lung	Lung Adenocarcinon	12.18	KRAS	
TCGA-67-4679-01	TCGA	Primary Tumor	Lung	Lung Adenocarcinon	12.17	KRAS	
TCGA-78-7167-01	TCGA	Primary Tumor	Lung	Lung Adenocarcinon	12.16	KRAS	
TCGA-75-7027-01	TCGA	Primary Tumor	Lung	Lung Adenocarcinon	12.16	KRAS	
TCGA-50-5932-01	TCGA	Primary Tumor	Lung	Lung Adenocarcinon	12.14	KRAS	
TCGA-05-4427-01	TCGA	Primary Tumor	Lung	Lung Adenocarcinon	12.14	KRAS	
TCGA-44-6145-01	TCGA	Primary Tumor	Lung	Lung Adenocarcinon	12.14	KRAS	
TCGA-44-A47A-01	TCGA	Primary Tumor	Lung	Lung Adenocarcinon	12.14	KRAS	
TCGA-05-4250-01	TCGA	Primary Tumor	Lung	Lung Adenocarcinon	12.13	KRAS	
TCGA-44-7671-01	TCGA	Primary Tumor	Lung	Lung Adenocarcinon	12.12	KRAS	
TCGA-05-4433-01	TCGA	Primary Tumor	Lung	Lung Adenocarcinon	12.1	KRAS	
TCGA-97-A4M0-01	TCGA	Primary Tumor	Lung	Lung Adenocarcinon	12.09	KRAS	
TCGA-MP-A4T7-01	TCGA	Primary Tumor	Lung	Lung Adenocarcinon	12.09	KRAS	
TCGA-50-8459-01	TCGA	Primary Tumor	Lung	Lung Adenocarcinon	12.08	KRAS	
TCGA-55-8203-01	TCGA	Primary Tumor	Lung	Lung Adenocarcinon	12.08	KRAS	
TCGA-86-8076-01	TCGA	Primary Tumor	Lung	Lung Adenocarcinon	12.08	KRAS	
TCGA-55-8302-01	TCGA	Primary Tumor	Lung	Lung Adenocarcinon	12.07	KRAS	
TCGA-95-7562-01	TCGA	Primary Tumor	Lung	Lung Adenocarcinon	12.07	KRAS	
TCGA-78-7145-01	TCGA	Primary Tumor	Lung	Lung Adenocarcinon	12.06	KRAS	
TCGA-NJ-A55O-01	TCGA	Primary Tumor	Lung	Lung Adenocarcinon	12.06	KRAS	
TCGA-44-7672-01	TCGA	Primary Tumor	Lung	Lung Adenocarcinon	12.05	KRAS	$ZNF24^{low}$
TCGA-99-8032-01	TCGA	Primary Tumor	Lung	Lung Adenocarcinon	12.04	KRAS	
TCGA-MP-A4TE-01	TCGA	Primary Tumor	Lung	Lung Adenocarcinon	12.04	KRAS	
TCGA-05-4418-01	TCGA	Primary Tumor	Lung	Lung Adenocarcinon	12.03	KRAS	

TCGA-86-8674-01	TCGA	Primary Tumor	Lung	Lung Adenocarcinon	12.03	KRAS
TCGA-55-7726-01	TCGA	Primary Tumor	Lung	Lung Adenocarcinon	12.02	KRAS
TCGA-50-5936-01	TCGA	Primary Tumor	Lung	Lung Adenocarcinon	12.01	KRAS
TCGA-78-7166-01	TCGA	Primary Tumor	Lung	Lung Adenocarcinon	11.97	KRAS
TCGA-05-4417-01	TCGA	Primary Tumor	Lung	Lung Adenocarcinon	11.96	KRAS
TCGA-MP-A4TK-01	TCGA	Primary Tumor	Lung	Lung Adenocarcinon	11.94	KRAS
TCGA-MP-A4TD-01	TCGA	Primary Tumor	Lung	Lung Adenocarcinon	11.93	KRAS
TCGA-MP-A4T4-01	TCGA	Primary Tumor	Lung	Lung Adenocarcinon	11.93	KRAS
TCGA-55-8094-01	TCGA	Primary Tumor	Lung	Lung Adenocarcinon	11.93	KRAS
TCGA-78-7160-01	TCGA	Primary Tumor	Lung	Lung Adenocarcinon	11.92	KRAS
TCGA-49-AARO-01	TCGA	Primary Tumor	Lung	Lung Adenocarcinon	11.91	KRAS
TCGA-91-6836-01	TCGA	Primary Tumor	Lung	Lung Adenocarcinon	11.88	KRAS
TCGA-55-6975-01	TCGA	Primary Tumor	Lung	Lung Adenocarcinon	11.88	KRAS
TCGA-NJ-A4YP-01	TCGA	Primary Tumor	Lung	Lung Adenocarcinon	11.88	KRAS
TCGA-05-4415-01	TCGA	Primary Tumor	Lung	Lung Adenocarcinon	11.85	KRAS
TCGA-73-7498-01	TCGA	Primary Tumor	Lung	Lung Adenocarcinon	11.84	KRAS
TCGA-21-1078-01	TCGA	Primary Tumor	Lung	Lung Squamous Cell	11.83	KRAS
TCGA-55-7283-01	TCGA	Primary Tumor	Lung	Lung Adenocarcinon	11.82	KRAS
TCGA-44-6776-01	TCGA	Primary Tumor	Lung	Lung Adenocarcinon	11.82	KRAS
TCGA-55-8616-01	TCGA	Primary Tumor	Lung	Lung Adenocarcinon	11.8	KRAS
TCGA-55-A490-01	TCGA	Primary Tumor	Lung	Lung Adenocarcinon	11.8	KRAS
TCGA-49-4510-01	TCGA	Primary Tumor	Lung	Lung Adenocarcinon	11.8	KRAS
TCGA-83-5908-01	TCGA	Primary Tumor	Lung	Lung Adenocarcinon	11.78	KRAS
TCGA-75-5126-01	TCGA	Primary Tumor	Lung	Lung Adenocarcinon	11.77	KRAS
TCGA-99-8028-01	TCGA	Primary Tumor	Lung	Lung Adenocarcinon	11.77	KRAS
TCGA-MP-A4TI-01	TCGA	Primary Tumor	Lung	Lung Adenocarcinon	11.76	KRAS
TCGA-95-7039-01	TCGA	Primary Tumor	Lung	Lung Adenocarcinon	11.76	KRAS
TCGA-64-5778-01	TCGA	Primary Tumor	Lung	Lung Adenocarcinon	11.72	KRAS
TCGA-55-7576-01	TCGA	Primary Tumor	Lung	Lung Adenocarcinon	11.71	KRAS
TCGA-63-6202-01	TCGA	Primary Tumor	Lung	Lung Squamous Cell	11.7	KRAS
TCGA-98-8023-01	TCGA	Primary Tumor	Lung	Lung Squamous Cell	11.68	KRAS
TCGA-55-7907-01	TCGA	Primary Tumor	Lung	Lung Adenocarcinon	11.66	KRAS
TCGA-21-5783-01	TCGA	Primary Tumor	Lung	Lung Squamous Cell	11.62	KRAS
TCGA-78-7161-01	TCGA	Primary Tumor	Lung	Lung Adenocarcinon	11.57	KRAS
TCGA-52-7812-01	TCGA	Primary Tumor	Lung	Lung Squamous Cell	11.57	KRAS
TCGA-55-7728-01	TCGA	Primary Tumor	Lung	Lung Adenocarcinon	11.56	KRAS
TCGA-62-A46R-01	TCGA	Primary Tumor	Lung	Lung Adenocarcinon	11.55	KRAS
TCGA-LA-A7SW-01	TCGA	Primary Tumor	Lung	Lung Squamous Cell	11.52	KRAS
TCGA-MP-A4T8-01	TCGA	Primary Tumor	Lung	Lung Adenocarcinon	11.51	KRAS
TCGA-97-7941-01	TCGA	Primary Tumor	Lung	Lung Adenocarcinon	11.42	KRAS
TCGA-49-AARQ-01	TCGA	Primary Tumor	Lung	Lung Adenocarcinon	11.38	KRAS
TCGA-MF-A522-01	TCGA	Primary Tumor	Lung	Lung Squamous Cell	11.35	KRAS
TCGA-J2-8194-01	TCGA	Primary Tumor	Lung	Lung Adenocarcinon	11.33	KRAS
TCGA-05-4395-01	TCGA	Primary Tumor	Lung	Lung Adenocarcinon	11.32	KRAS
TCGA-49-4506-01	TCGA	Primary Tumor	Lung	Lung Adenocarcinon	11.2	KRAS
TCGA-64-5775-01	TCGA	Primary Tumor	Lung	Lung Adenocarcinon	11.16	KRAS
TCGA-97-8179-01	TCGA	Primary Tumor	Lung	Lung Adenocarcinon	11.12	KRAS
TCGA-50-5051-01	TCGA	Primary Tumor	Lung	Lung Adenocarcinon	11.01	KRAS
TCGA-O2-A52Q-01	TCGA	Primary Tumor	Lung	Lung Squamous Cell	10.89	KRAS
TCGA-97-8176-01	TCGA	Primary Tumor	Lung	Lung Adenocarcinon	10.79	KRAS
TCGA-73-4670-01	TCGA	Primary Tumor	Lung	Lung Adenocarcinon	10.79	KRAS
