# Supplementary material for: In vivo genome-wide CRISPR screening identifies ZNF24 as a negative NF-κB modulator in lung cancer
Source: Cell Biosci. 2022 Dec 1;12:193. doi: 10.1186/s13578-022-00933-0 (PMC9717477; doi:10.1186/s13578-022-00933-0)
Supplement: Supplementary file 3 — Additional file 3: Table S3. Clinical database from UCSC Xena(http://xena.ucsc.edu/) analysis ofZNF24 expression using the median value as a cutoff. Analysis thepercentage patients with low expression of ZNF24 inpatients with EGFR mutations. [file 13578_2022_933_MOESM3_ESM.pdf]

Table S3

Clinical database from UCSC Xena(http://xena.ucsc.edu/) analysis of ZNF24 expression using the median value as a cutoff. Analysis the percentage patients with low expression of ZNF24 in patients with EGFR mutations.

sample	study	sample_type	primary _site	primary disease or tissue	ZNF24 expression	EGFR mutation	
TCGA-55-6968-01	TCGA	Primary Tumor	Lung	Lung Adenocarcinoma	13.93	EGFR	
TCGA-86-8075-01	TCGA	Primary Tumor	Lung	Lung Adenocarcinoma	13.56	EGFR	
TCGA-86-8055-01	TCGA	Primary Tumor	Lung	Lung Adenocarcinoma	13.5	EGFR	
TCGA-46-3769-01	TCGA	Primary Tumor	Lung	Lung Squamous Cell Ca	13.28	EGFR	
TCGA-55-7570-01	TCGA	Primary Tumor	Lung	Lung Adenocarcinoma	13.25	EGFR	
TCGA-44-6147-01	TCGA	Primary Tumor	Lung	Lung Adenocarcinoma	13.1	EGFR	
TCGA-44-6147-01	TCGA	Primary Tumor	Lung	Lung Adenocarcinoma	13.1	EGFR	
TCGA-55-8096-01	TCGA	Primary Tumor	Lung	Lung Adenocarcinoma	13.01	EGFR	
TCGA-50-6673-01	TCGA	Primary Tumor	Lung	Lung Adenocarcinoma	12.97	EGFR	
TCGA-66-2789-01	TCGA	Primary Tumor	Lung	Lung Squamous Cell Ca	12.91	EGFR	
TCGA-50-5944-01	TCGA	Primary Tumor	Lung	Lung Adenocarcinoma	12.83	EGFR	
TCGA-67-3771-01	TCGA	Primary Tumor	Lung	Lung Adenocarcinoma	12.8	EGFR	
TCGA-86-8668-01	TCGA	Primary Tumor	Lung	Lung Adenocarcinoma	12.79	EGFR	
TCGA-86-A4P7-0	TCGA	Primary Tumor	Lung	Lung Adenocarcinoma	12.79	EGFR	
TCGA-55-7573-01	TCGA	Primary Tumor	Lung	Lung Adenocarcinoma	12.78	EGFR	
TCGA-55-7573-01	TCGA	Primary Tumor	Lung	Lung Adenocarcinoma	12.78	EGFR	
TCGA-44-2661-01	TCGA	Primary Tumor	Lung	Lung Adenocarcinoma	12.66	EGFR	
TCGA-05-5423-01	TCGA	Primary Tumor	Lung	Lung Adenocarcinoma	12.56	EGFR	
TCGA-05-5423-01	TCGA	Primary Tumor	Lung	Lung Adenocarcinoma	12.56	EGFR	
TCGA-62-8402-01	TCGA	Primary Tumor	Lung	Lung Adenocarcinoma	12.56	EGFR	
TCGA-22-4609-01	TCGA	Primary Tumor	Lung	Lung Squamous Cell Ca	12.54	EGFR	
TCGA-55-8206-01	TCGA	Primary Tumor	Lung	Lung Adenocarcinoma	12.51	EGFR	
TCGA-66-2742-01	TCGA	Primary Tumor	Lung	Lung Squamous Cell Ca	12.5	EGFR	
TCGA-55-6980-01	TCGA	Primary Tumor	Lung	Lung Adenocarcinoma	12.45	EGFR	
TCGA-64-1681-01	TCGA	Primary Tumor	Lung	Lung Adenocarcinoma	12.45	EGFR	
TCGA-56-8304-01	TCGA	Primary Tumor	Lung	Lung Squamous Cell Ca	12.45	EGFR	
TCGA-91-6835-01	TCGA	Primary Tumor	Lung	Lung Adenocarcinoma	12.42	EGFR	
TCGA-93-A4JP-01	TCGA	Primary Tumor	Lung	Lung Adenocarcinoma	12.4	EGFR	ZNF24^{high}
TCGA-69-7765-01	TCGA	Primary Tumor	Lung	Lung Adenocarcinoma	12.39	EGFR	
TCGA-86-8074-01	TCGA	Primary Tumor	Lung	Lung Adenocarcinoma	12.39	EGFR	
TCGA-85-A50M-0	TCGA	Primary Tumor	Lung	Lung Squamous Cell Ca	12.39	EGFR	
TCGA-97-8547-01	TCGA	Primary Tumor	Lung	Lung Adenocarcinoma	12.38	EGFR	
TCGA-86-A4JF-01	TCGA	Primary Tumor	Lung	Lung Adenocarcinoma	12.37	EGFR	
TCGA-85-A4JB-0	TCGA	Primary Tumor	Lung	Lung Squamous Cell Ca	12.37	EGFR	
TCGA-05-4382-01	TCGA	Primary Tumor	Lung	Lung Adenocarcinoma	12.34	EGFR	
TCGA-05-4382-01	TCGA	Primary Tumor	Lung	Lung Adenocarcinoma	12.34	EGFR	
TCGA-MP-A4T9-0	TCGA	Primary Tumor	Lung	Lung Adenocarcinoma	12.33	EGFR	
TCGA-55-A48Z-0	TCGA	Primary Tumor	Lung	Lung Adenocarcinoma	12.29	EGFR	
TCGA-97-A4M7-0	TCGA	Primary Tumor	Lung	Lung Adenocarcinoma	12.29	EGFR	
TCGA-J2-8192-01	TCGA	Primary Tumor	Lung	Lung Adenocarcinoma	12.28	EGFR	
TCGA-97-A4M6-0	TCGA	Primary Tumor	Lung	Lung Adenocarcinoma	12.27	EGFR	
TCGA-49-4501-01	TCGA	Primary Tumor	Lung	Lung Adenocarcinoma	12.25	EGFR	
TCGA-55-6981-01	TCGA	Primary Tumor	Lung	Lung Adenocarcinoma	12.25	EGFR	
TCGA-50-5933-01	TCGA	Primary Tumor	Lung	Lung Adenocarcinoma	12.21	EGFR	
TCGA-85-8287-01	TCGA	Primary Tumor	Lung	Lung Squamous Cell Ca	12.21	EGFR	
TCGA-43-A56U-0	TCGA	Primary Tumor	Lung	Lung Squamous Cell Ca	12.2	EGFR	

TCGA-44-5645-01	TCGA	Primary Tumor Lung	Lung Adenocarcinoma	12.19	EGFR	
TCGA-MP-A4SW-	TCGA	Primary Tumor Lung	Lung Adenocarcinoma	12.19	EGFR	
TCGA-05-4410-01	TCGA	Primary Tumor Lung	Lung Adenocarcinoma	12.14	EGFR	
TCGA-50-6591-01	TCGA	Primary Tumor Lung	Lung Adenocarcinoma	12.14	EGFR	
TCGA-50-6591-01	TCGA	Primary Tumor Lung	Lung Adenocarcinoma	12.14	EGFR	
TCGA-55-8506-01	TCGA	Primary Tumor Lung	Lung Adenocarcinoma	12.14	EGFR	
TCGA-MP-A4T6-(	TCGA	Primary Tumor Lung	Lung Adenocarcinoma	12.12	EGFR	
TCGA-67-6217-01	TCGA	Primary Tumor Lung	Lung Adenocarcinoma	12.09	EGFR	
TCGA-97-8172-01	TCGA	Primary Tumor Lung	Lung Adenocarcinoma	12.05	EGFR	
TCGA-77-8156-01	TCGA	Primary Tumor Lung	Lung Squamous Cell Ca	12.02	EGFR	
TCGA-69-7760-01	TCGA	Primary Tumor Lung	Lung Adenocarcinoma	12.01	EGFR	
TCGA-78-7158-01	TCGA	Primary Tumor Lung	Lung Adenocarcinoma	12.01	EGFR	
TCGA-55-A57B-0	TCGA	Primary Tumor Lung	Lung Adenocarcinoma	11.97	EGFR	
TCGA-70-6722-01	TCGA	Primary Tumor Lung	Lung Squamous Cell Ca	11.96	EGFR	
TCGA-70-6722-01	TCGA	Primary Tumor Lung	Lung Squamous Cell Ca	11.96	EGFR	
TCGA-95-7947-01	TCGA	Primary Tumor Lung	Lung Adenocarcinoma	11.95	EGFR	
TCGA-95-7947-01	TCGA	Primary Tumor Lung	Lung Adenocarcinoma	11.95	EGFR	
TCGA-85-7950-01	TCGA	Primary Tumor Lung	Lung Squamous Cell Ca	11.94	EGFR	
TCGA-05-4402-01	TCGA	Primary Tumor Lung	Lung Adenocarcinoma	11.93	EGFR	
TCGA-05-4402-01	TCGA	Primary Tumor Lung	Lung Adenocarcinoma	11.93	EGFR	
TCGA-71-8520-01	TCGA	Primary Tumor Lung	Lung Adenocarcinoma	11.93	EGFR	
TCGA-71-8520-01	TCGA	Primary Tumor Lung	Lung Adenocarcinoma	11.93	EGFR	
TCGA-38-4627-01	TCGA	Primary Tumor Lung	Lung Adenocarcinoma	11.92	EGFR	
TCGA-38-4627-01	TCGA	Primary Tumor Lung	Lung Adenocarcinoma	11.92	EGFR	
TCGA-97-A4M1-(	TCGA	Primary Tumor Lung	Lung Adenocarcinoma	11.89	EGFR	
TCGA-L9-A50W-(	TCGA	Primary Tumor Lung	Lung Adenocarcinoma	11.89	EGFR	
TCGA-L9-A50W-(	TCGA	Primary Tumor Lung	Lung Adenocarcinoma	11.89	EGFR	
TCGA-75-7025-01	TCGA	Primary Tumor Lung	Lung Adenocarcinoma	11.88	EGFR	
TCGA-85-7844-01	TCGA	Primary Tumor Lung	Lung Squamous Cell Ca	11.88	EGFR	
TCGA-62-A46U-0	TCGA	Primary Tumor Lung	Lung Adenocarcinoma	11.86	EGFR	
TCGA-55-8616-01	TCGA	Primary Tumor Lung	Lung Adenocarcinoma	11.8	EGFR	
TCGA-38-4628-01	TCGA	Primary Tumor Lung	Lung Adenocarcinoma	11.79	EGFR	
TCGA-95-7039-01	TCGA	Primary Tumor Lung	Lung Adenocarcinoma	11.76	EGFR	
TCGA-50-6595-01	TCGA	Primary Tumor Lung	Lung Adenocarcinoma	11.75	EGFR	
TCGA-50-6595-01	TCGA	Primary Tumor Lung	Lung Adenocarcinoma	11.75	EGFR	
TCGA-78-7147-01	TCGA	Primary Tumor Lung	Lung Adenocarcinoma	11.75	EGFR	
TCGA-78-7147-01	TCGA	Primary Tumor Lung	Lung Adenocarcinoma	11.75	EGFR	$ZNF24^{low}$
TCGA-78-7147-01	TCGA	Primary Tumor Lung	Lung Adenocarcinoma	11.75	EGFR	
TCGA-78-7155-01	TCGA	Primary Tumor Lung	Lung Adenocarcinoma	11.7	EGFR	
TCGA-78-7155-01	TCGA	Primary Tumor Lung	Lung Adenocarcinoma	11.7	EGFR	
TCGA-78-7155-01	TCGA	Primary Tumor Lung	Lung Adenocarcinoma	11.7	EGFR	
TCGA-56-7222-01	TCGA	Primary Tumor Lung	Lung Squamous Cell Ca	11.64	EGFR	
TCGA-97-8552-01	TCGA	Primary Tumor Lung	Lung Adenocarcinoma	11.63	EGFR	
TCGA-97-8177-01	TCGA	Primary Tumor Lung	Lung Adenocarcinoma	11.58	EGFR	
TCGA-22-4601-01	TCGA	Primary Tumor Lung	Lung Squamous Cell Ca	11.54	EGFR	
TCGA-49-6743-01	TCGA	Primary Tumor Lung	Lung Adenocarcinoma	11.52	EGFR	
TCGA-49-4490-01	TCGA	Primary Tumor Lung	Lung Adenocarcinoma	11.51	EGFR	
TCGA-49-4494-01	TCGA	Primary Tumor Lung	Lung Adenocarcinoma	11.5	EGFR	
TCGA-49-4494-01	TCGA	Primary Tumor Lung	Lung Adenocarcinoma	11.5	EGFR	
TCGA-44-A4SU-0	TCGA	Primary Tumor Lung	Lung Adenocarcinoma	11.49	EGFR	
TCGA-44-A4SU-0	TCGA	Primary Tumor Lung	Lung Adenocarcinoma	11.49	EGFR	
TCGA-67-3770-01	TCGA	Primary Tumor Lung	Lung Adenocarcinoma	11.48	EGFR	
TCGA-50-5066-01	TCGA	Primary Tumor Lung	Lung Adenocarcinoma	11.42	EGFR	
TCGA-60-2708-01	TCGA	Primary Tumor Lung	Lung Squamous Cell Ca	11.42	EGFR	

TCGA-86-8280-01	TCGA	Primary Tumor	Lung	Lung Adenocarcinoma	11.39	EGFR
TCGA-62-8394-01	TCGA	Primary Tumor	Lung	Lung Adenocarcinoma	11.38	EGFR
TCGA-60-2698-01	TCGA	Primary Tumor	Lung	Lung Squamous Cell Ca	11.37	EGFR
TCGA-97-8171-01	TCGA	Primary Tumor	Lung	Lung Adenocarcinoma	11.36	EGFR
TCGA-85-7697-01	TCGA	Primary Tumor	Lung	Lung Squamous Cell Ca	11.34	EGFR
TCGA-38-4629-01	TCGA	Primary Tumor	Lung	Lung Adenocarcinoma	11.2	EGFR
TCGA-38-6178-01	TCGA	Primary Tumor	Lung	Lung Adenocarcinoma	11.2	EGFR
TCGA-75-6212-01	TCGA	Primary Tumor	Lung	Lung Adenocarcinoma	11.16	EGFR
TCGA-66-2763-01	TCGA	Primary Tumor	Lung	Lung Squamous Cell Ca	11.12	EGFR
TCGA-75-6207-01	TCGA	Primary Tumor	Lung	Lung Adenocarcinoma	10.78	EGFR
TCGA-18-3417-01	TCGA	Primary Tumor	Lung	Lung Squamous Cell Ca	10.7	EGFR
TCGA-33-4566-01	TCGA	Primary Tumor	Lung	Lung Squamous Cell Ca	10.6	EGFR
