# Supplementary material for: In vivo genome-wide CRISPR screening identifies ZNF24 as a negative NF-κB modulator in lung cancer
Source: Cell Biosci. 2022 Dec 1;12:193. doi: 10.1186/s13578-022-00933-0 (PMC9717477; doi:10.1186/s13578-022-00933-0)
Supplement: Supplementary file 4 — Additional file 4: Figure S1. ZNF24 is an essential tumor suppressor in lung cancer. (A) Western blot analysis of protein levels of Cas9 in EKVX cells. EKVX cells were infected with virus encoding Cas9, and selected with blasticidin for two weeks. Monoclone was picked to generate a stable cell line (EKVX-Cas9). (B) Scatter diagram showing genes targeted by sgRNAs that were differentially depleted or enriched in EKVX-Cas9 cells. (C) KEGG analysis of the top enriched 230 genes in genome-wide screening through CRISPR/Cas9. (D) RT-qPCR detect the knock-down efficiency of ZNF24, NR3C2, CST4, ARHGDIG and CRYBB3. RNA was extracted from engineered EKVX cells. Expression of the ZNF24, NR3C2, CST4, ARHGDIG and CRYBB3 were quantified through RT-qPCR. (E-I) Impact of expression level of ZNF24, NR3C2, CST4, ARHGDIG and CRYBB3 on proliferation of EKVX cells. Engineered EKVX cells were seeded in 96-well plates and cultured for 4 days. Cell viability was analyzed with CCK8. shGFP as control knockdown. (J) Comparison of protein levels of ZNF24 in tumors, para-tumoral lung tissues and lung cancer cell lines. Expression level of ZNF24 was checked through western blotting analysis on tumors, para-tumoral lung tissues (upper panel). Evaluation of ZNF24 expression with RT-qPCR in various lung cancer cell lines (lower panel). RNA was extracted from various lung cancer cell lines. Expression of the ZNF24 were quantified through RT-qPCR. Bars are represented as mean ± SEM of the indicated number (n) of repeats. *P<0.05, **P<0.01, and ***P<0.001 by Student’s t-test. Figure S2. ZNF24 is an essential tumor suppressor in lung cancer. (A) Western Blot evaluation of ZNF24 expression induced by Dox (1 μg/mL) for 48 h in A549i and Hop62i cells. (B) Impact of ZNF24 expression level on proliferation of A549i and Hop62i cells. A549, Hop62 infected with lenti-virus for expressing ZNF24 (designated A549i, Hop62i). A549i, Hop62i (1000 cells) were respectively inoculated in 96-well plates and cultured wit [file 13578_2022_933_MOESM4_ESM.docx]

**Title:** *In vivo* genome-wide CRISPR screening identifies ZNF24 as a negative NF-κB modulator in lung cancer

**Running Title:** ZNF24 inhibits transcription of *P65*

Lu Liu^1^*, Yuxi Lei^1^*, Wensheng Chen^1^*, Qian Zhou^1^*^#^, Zongyao Zheng^1^, Guandi Zeng^1^, Wanting Liu^1^, Pengju Feng^2^, Zhiyi Zhang^1^, Lei Yu^3#^, Liang Chen^1#^

1 MOE Key Laboratory of Tumor Molecular Biology and Key Laboratory of Functional Protein Research of Guangdong Higher Education Institutes, Institute of Life and Health Engineering, College of Life Science and Technology, Jinan University, Guangzhou 510632, China.

2 Department of Chemistry, Jinan University, Guangzhou 510632, China.

3 Beijing Tongren Hospital, Capital Medical University, Beijing 100730, China.

* These authors contributed equally to this work

# Corresponding Author:

Liang Chen, Email: [chenliang@jnu.edu.cn](mailto:chenliang@jnu.edu.cn).

Lei Yu, E-mail: yulei1118@sohu.com

Qian Zhou, E-mail: zhouqian_whu@163.com

**Key words: Lung cancer; Tumor suppressor gene; ZNF24; NF-κB; Combination therapy**

**
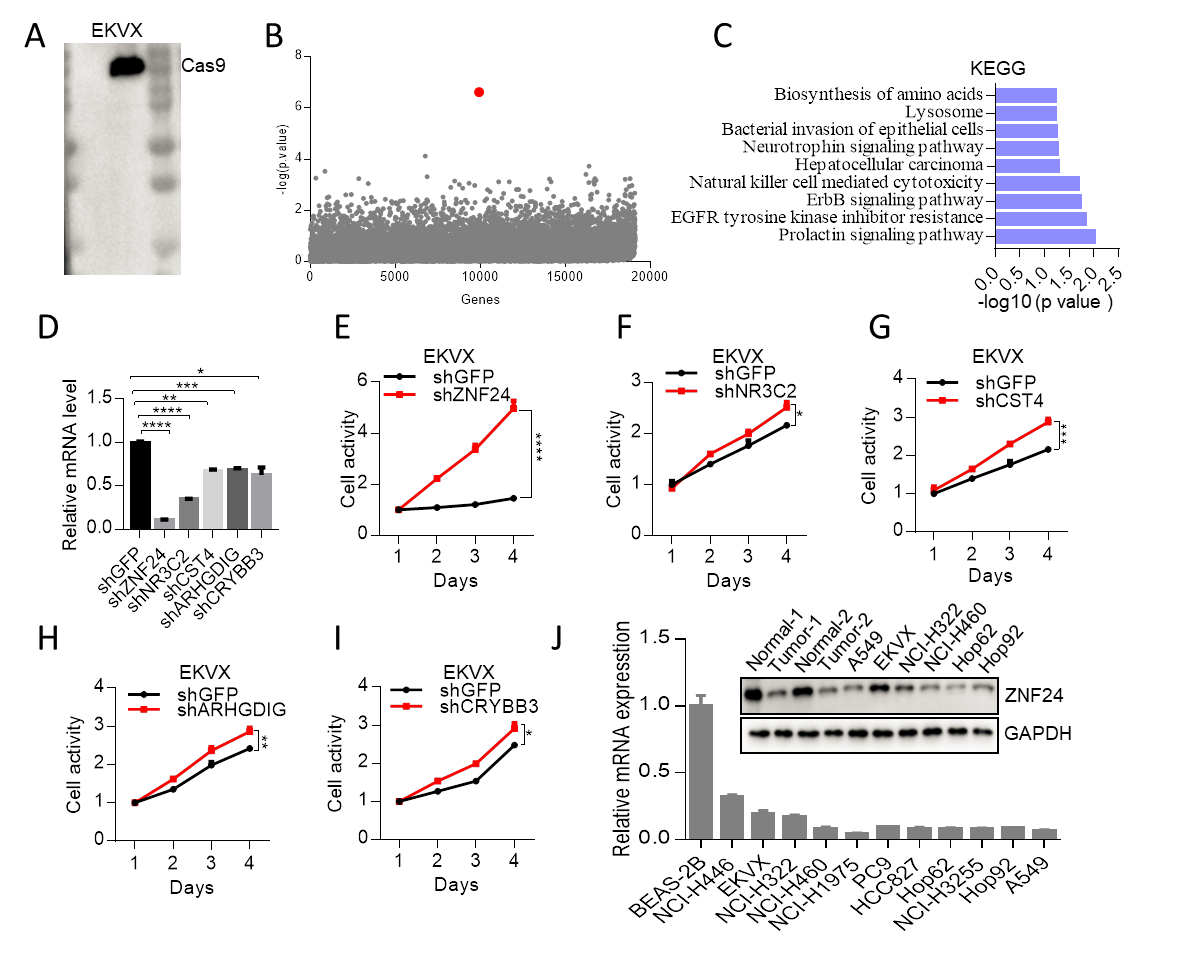
Figure S1. *ZNF24* is an essential tumor suppressor in lung cancer. (A)** Western blot analysis of protein levels of Cas9 in EKVX cells. EKVX cells were infected with virus encoding Cas9, and selected with blasticidin for two weeks. Monoclone was picked to generate a stable cell line (EKVX-Cas9). **(B)** Scatter diagram showing genes targeted by sgRNAs that were differentially depleted or enriched in EKVX-Cas9 cells. **(C)** KEGG analysis of the top enriched 230 genes in genome-wide screening through CRISPR/Cas9. **(D)** RT-qPCR detect the knock-down efficiency of *ZNF24*, *NR3C2*, *CST4*, *ARHGDIG* and *CRYBB3*. RNA was extracted from engineered EKVX cells. Expression of the *ZNF24*, *NR3C2*, *CST4*, *ARHGDIG* and *CRYBB3* were quantified through RT-qPCR. **(E-I)** Impact of expression level of ZNF24, NR3C2, CST4, ARHGDIG and CRYBB3 on proliferation of EKVX cells. Engineered EKVX cells were seeded in 96-well plates and cultured for 4 days. Cell viability was analyzed with CCK8. shGFP as control knockdown. **(J)** Comparison of protein levels of ZNF24 in tumors, para-tumoral lung tissues and lung cancer cell lines. Expression level of ZNF24 was checked through western blotting analysis on tumors, para-tumoral lung tissues (upper panel). Evaluation of ZNF24 expression with RT-qPCR in various lung cancer cell lines (lower panel). RNA was extracted from various lung cancer cell lines. Expression of the ZNF24 were quantified through RT-qPCR. Bars are represented as mean ± SEM of the indicated number (n) of repeats. *P<0.05, **P<0.01, and ***P<0.001 by Student’s t-test.


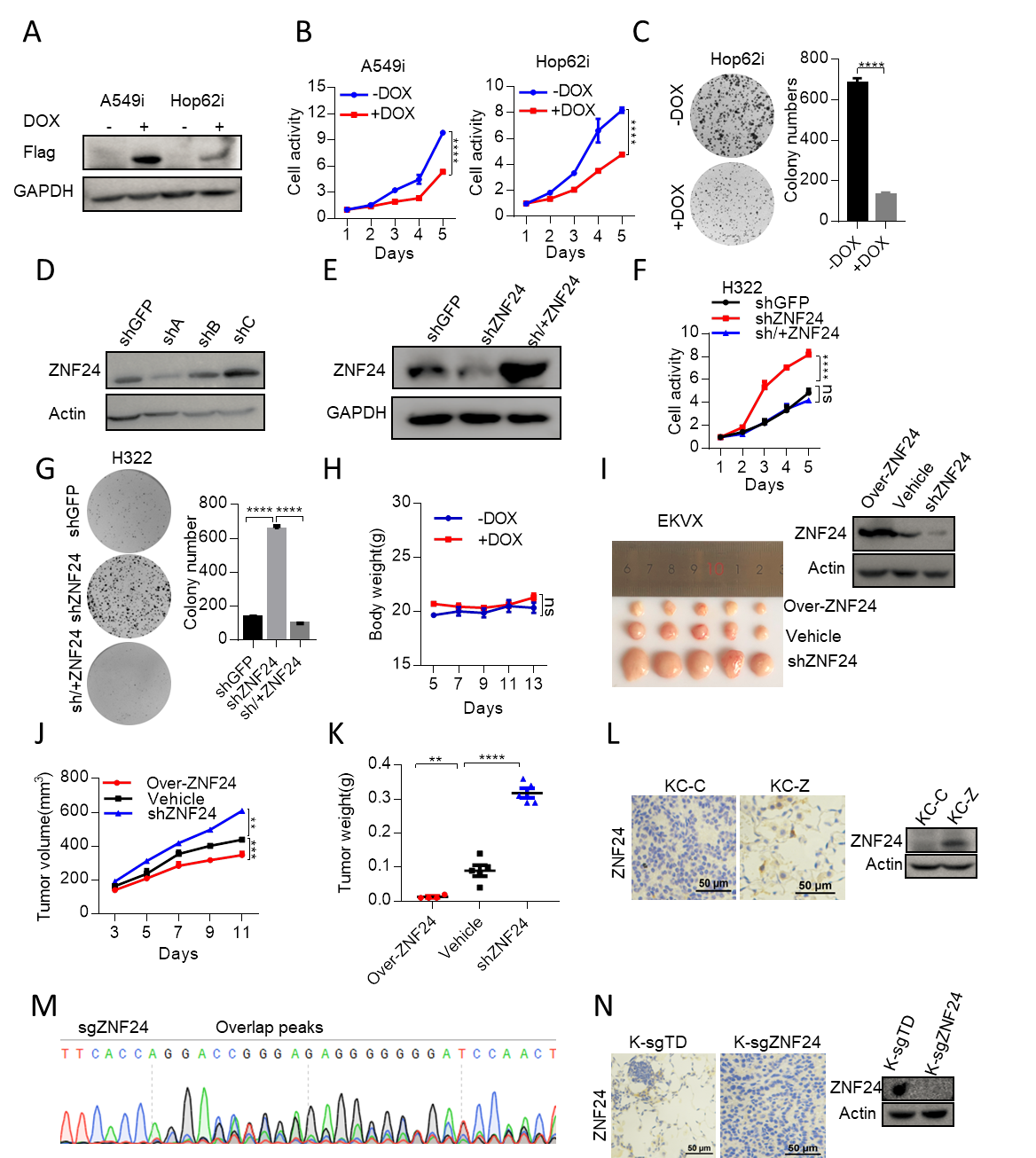


**Figure S2. *ZNF24* is an essential tumor suppressor in lung cancer. (A)** Western Blot evaluation of ZNF24 expression induced by Dox (1 μg/mL) for 48 h in A549i and Hop62i cells. **(B)** Impact of ZNF24 expression level on proliferation of A549i and Hop62i cells. A549, Hop62 infected with lenti-virus for expressing ZNF24 (designated A549i, Hop62i). A549i, Hop62i (1000 cells) were respectively inoculated in 96-well plates and cultured with 1 μg/mL of Dox for 5 days. Cell viability was analyzed with CCK8. **(C)** Impact of ZNF24 expression level on colony forming ability of Hop62i cells. Hop62i (1000 cells) were seeded in 6-well plates and treated with Dox (1 μg/mL) for 2 weeks before quantification for colonies. Left: representative pictures. Right: statistics of colony number. **(D)** Validation of knockdown efficiency of three ZNF24 shRNAs. 293T cells were co-transfected with pCDNA3.1-ZNF24 and one of three plasmids encoding shZNF24s. Expression level of ZNF24 was analyzed through Western Blot. shRNA A exhibited the highest knockdown efficiency. **(E)** Western blot detecting the knockdown and replenishing efficiency of ZNF24 in EKVX cells. **(F)** Effect of ZNF24 knockdown or re-expression on proliferation of H322 cells. 1000 engineered H322 cells were seeded in 96-well plates and cultured for 5 days. Cell viability was analyzed with CCK8. shGFP as control knockdown; shZNF24 for ZNF24 knockdown; sh/+ZNF24 for ZNF24 re-expression in ZNF24 knockdown cells. **(G)** Impact of ZNF24 knockdown or re-expression on 2-D colony formation ability of H322 cells. 1000 engineered H322 cells were respectively inoculated in 6-well plates and cultured for 2 weeks before quantification for colonies. Left: representative pictures. Right: statistics of colony number. **(H)** Nontoxic effect of Dox on the weight of nude mice. **(I)** Impact of ZNF24 on EKVX xenografted tumors. Cells (2×10^6^) were inoculated into nude mice for tumor growth for 14 days. Tumor volume is recorded every 2 days. A representative image of the tumors at the end of the experiments is shown. **(J-K)** The tumor growth or weight were monitored of **(I). (L)** Expression of ZNF24 in lungs of KC-C; KC-Z transgenic mice revealed through IHC staining (left) and western blot (right). **(M)** Knockout efficiency of ZNF24 by lenti-virus encoded CRISPR confirmed through Sanger sequencing. **(N)** IHC staining of ZNF24 in mouse section of K-sgTD mice and K-sgZNF24 mice (left). Western blot detecting the knockout efficiency of ZNF24 in K-sgTD mice and K-sgZNF24 mice (right). Bars are represented as mean ± SEM of the indicated number (n) of repeats. *P<0.05, **P<0.01, and ***P<0.001 by Student’s t-test.


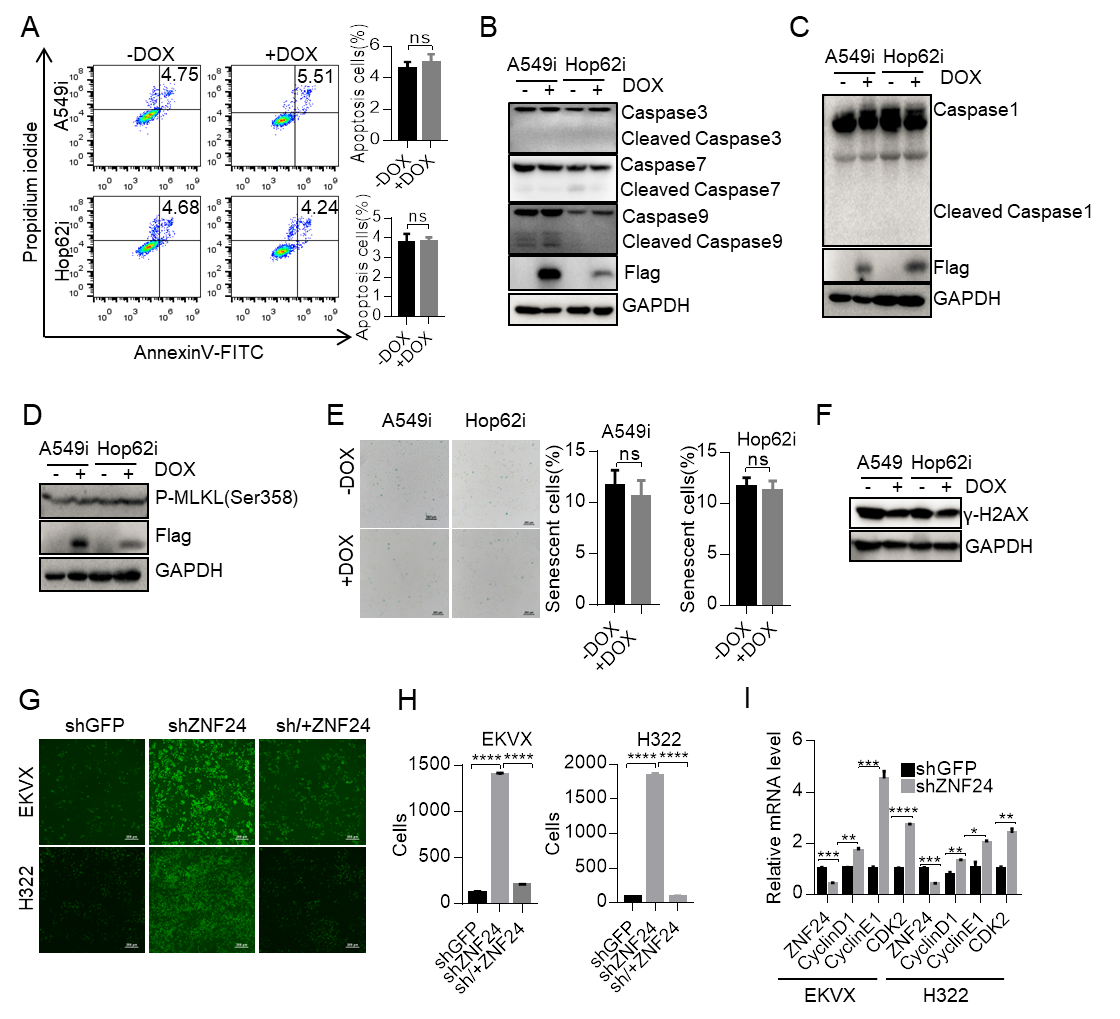
**Figure S3. Ectopic expression of ZNF24 induces cell-cycle arrest of lung cancer cell. (A)** Impact of ZNF24 expression on cell apoptosis in A549i, Hop62i cells. A549i, Hop62i cells were treated with Dox (1 μg/mL) for 48 h. Flow cytometry detection of the effect of overexpression of ZNF24 on cell apoptosis. **(B)** Western blot evaluation of expression of apoptosis-related proteins. A549i, Hop62i cells were treated with Dox (1 μg/mL) for 48 h. The cells were harvested for immunoblot analysis with indicated antibodies. **(C)** Impact of ZNF24 expression on cell pyroptosis in A549i, Hop62i cells. A549i, Hop62i cells were treated with Dox (1 μg/mL) for 48 h. The cells were harvested for immunoblot analysis with indicated antibodies. **(D)** Impact of ZNF24 expression on cell necroptosis in A549i, Hop62i cells. A549i, Hop62i cells were treated with Dox (1 μg/mL) for 48 h. The cells were harvested for immunoblot analysis with indicated antibodies. **(E)** Impact of ZNF24 expression on senescence in A549i and Hop62i cells. A549i and Hop62i cells were treated with Dox (1 μg/mL) for 48 h before β-galactosidase staining. Representative pictures (left) and statistics (right). **(F)** Impact of ZNF24 expression on DNA damage in A549i, Hop62i cells. A549i and Hop62i cells were treated with Dox (1 μg/mL) for 48 h. Western Blot detected γ-H2AX in A549i and Hop62i cells. **(G)** Impact of ZNF24 expression on proliferation of EKVX and H322 cells. Cells were cultured in media containing 10 μM of Edu dye. Cell proliferation was evaluated by checking the incorporation of Edu through fluorescent microscopy. **(H)** Statistics of **(G). (I)** RT-qPCR analysis of impact of ZNF24 expression on *CDK2*, *Cyclin D1* and *Cyclin E1* expression in EKVX, H322 cells. Bars are represented as mean ± SEM of the indicated number (n) of repeats. *P<0.05, **P<0.01, and ***P<0.001 by Student’s t-test.


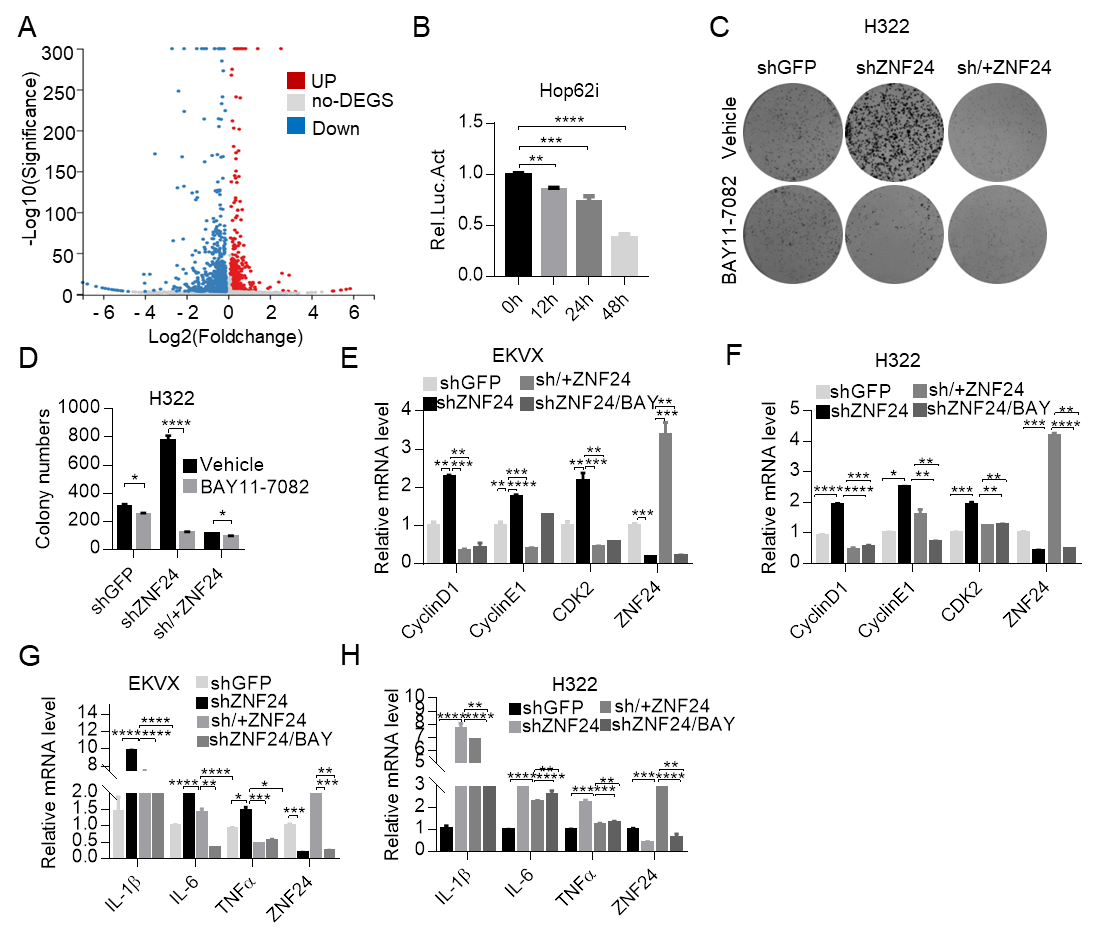
**Figure S4. ZNF24 induces cell cycle arrest through NF-κB signaling pathways. (A)** The volcano map representation of impact of ZNF24 expression on transcriptome of A549i cells. A549i cells were treated with 1 μg /mL of Dox for 48 h. RNAs were isolated and subjected to RNA-sequencing. **(B)** Impact of ZNF24 expression on NF-κB reporter in Hop62i cells. Hop62i cells were transfected with the NF-κB reporter. Luciferase assays were performed 12, 24 and 48 h after inducing with Dox (1 μg/mL). **(C)** Knock-down of ZNF24 in H322 cells sensitized to the inhibitors of NF-κB. H322-shGFP, H322-shZNF24, H322-sh/+ZNF24 cells (1000) were seeded in six-well plates and treated with DMSO or NF-κB inhibitor (BAY11-7082, 2 μM) for 2 weeks. Cells were stained with 0.5% crystal violet. **(D)** Statistics of **(C). (E**-**F)** Impact of NF-κB inhibitor on expression of cycle-related proteins by lung cancer cells. RT-qPCR detection of expression on cycle related genes. After treatment of EKVX-shZNF24 and H322-shZNF24 cells with BAY11-7082 (2 μM) for 24 h. E. data on EKVX-shZNF24 cells. F. data on H322-shZNF24 cells. **(G-H)** Impact of NF-κB inhibitor on transcription of target genes (*IL-1β*, *IL-6*, *TNFα*) by EKVX-shZNF24 and H322-shZNF24 cells. RT-qPCR analysis of expression of related genes after treatment of EKVX-shZNF24 and H322-shZNF24 cells with BAY11-7082 (2 μM) for 24 h. G. data on EKVX-shZNF24 cells. H. data on H322-shZNF24 cells. Bars are represented as mean ± SEM of the indicated number (n) of repeats. *P<0.05, **P<0.01, and ***P<0.001 by Student’s t-test.


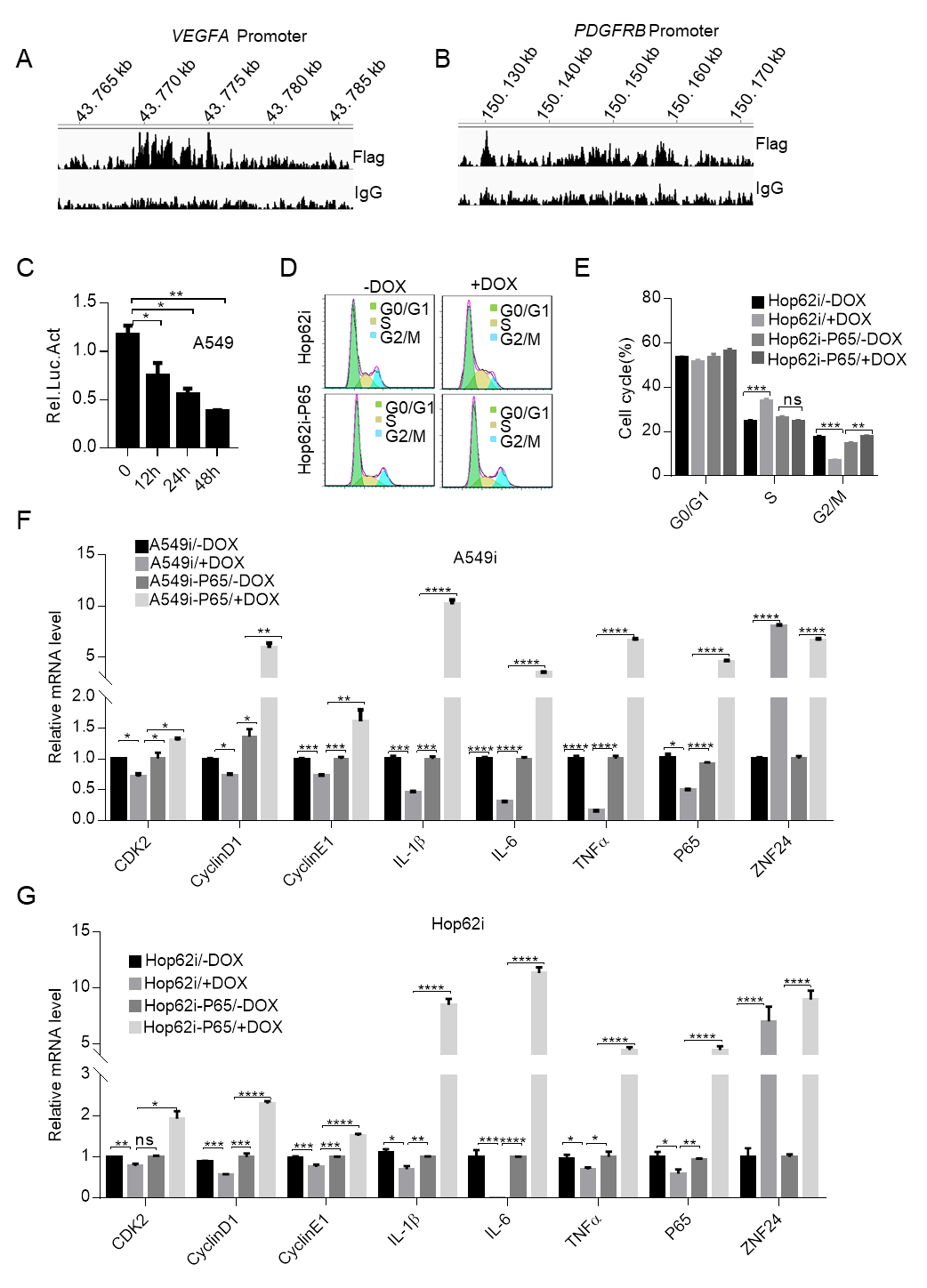
**Figure S5. ZNF24 binds *P65* promoter to negatively regulate its expression. (A-B)** Genome-wide analysis of ZNF24 binding sites in lung cancer cells through ChIP-seq analysis. A549i cells were treated with Dox (1 μg/mL) for 48 h. ChIP-seq was performed on DNA samples enriched for the Flag antibody. ZNF24 bound the promoter region of *VEGFA* **(A)** and *PDGFRB* **(B)** genes. **(C)** Impact of ZNF24 expression on activity of *P65*. *P65* promoter region was cloned into pGL3-Luciferase plasmid (designated pGL3-*P65*-Luciferase). The construct (0.5 μg) was transfected into A549 cells together with ZNF24 expression plasmid (0.5 mg). Luciferase activity was monitored 12, 24 and 48 h later. **(D)** Impact of P65 expression of cell cycle in Hop62i and Hop62i-P65 cells. Hop62i and Hop62i-P65 cells treated with Dox (1 μg/mL) for 48 h. Cell cycle were determined through FACS analysis of DNA contents revealed by propidium Iodide (PI) staining. Results are represented as percent of cell population in G0/G1, S and G2/M phases of the cell cycle. **(E)** Statistics of **(D).** **(F-G)** Impact of P65 expression on cyclin-associated genes and NF-κB target genes in A549i-P65 and Hop62i-P65 cells. A549i-P65 and Hop62i-P65 cells were treated with Dox (1 μg/mL) for 48 h. RNA was extracted from cells. Expression of the indicated genes were quantified through RT-qPCR. F. data on A549i cells. G. data on Hop62i cells. Bars are represented as mean ± SEM of the indicated number (n) of repeats. *P<0.05, **P<0.01, and ***P<0.001 by Student’s t-test.

**
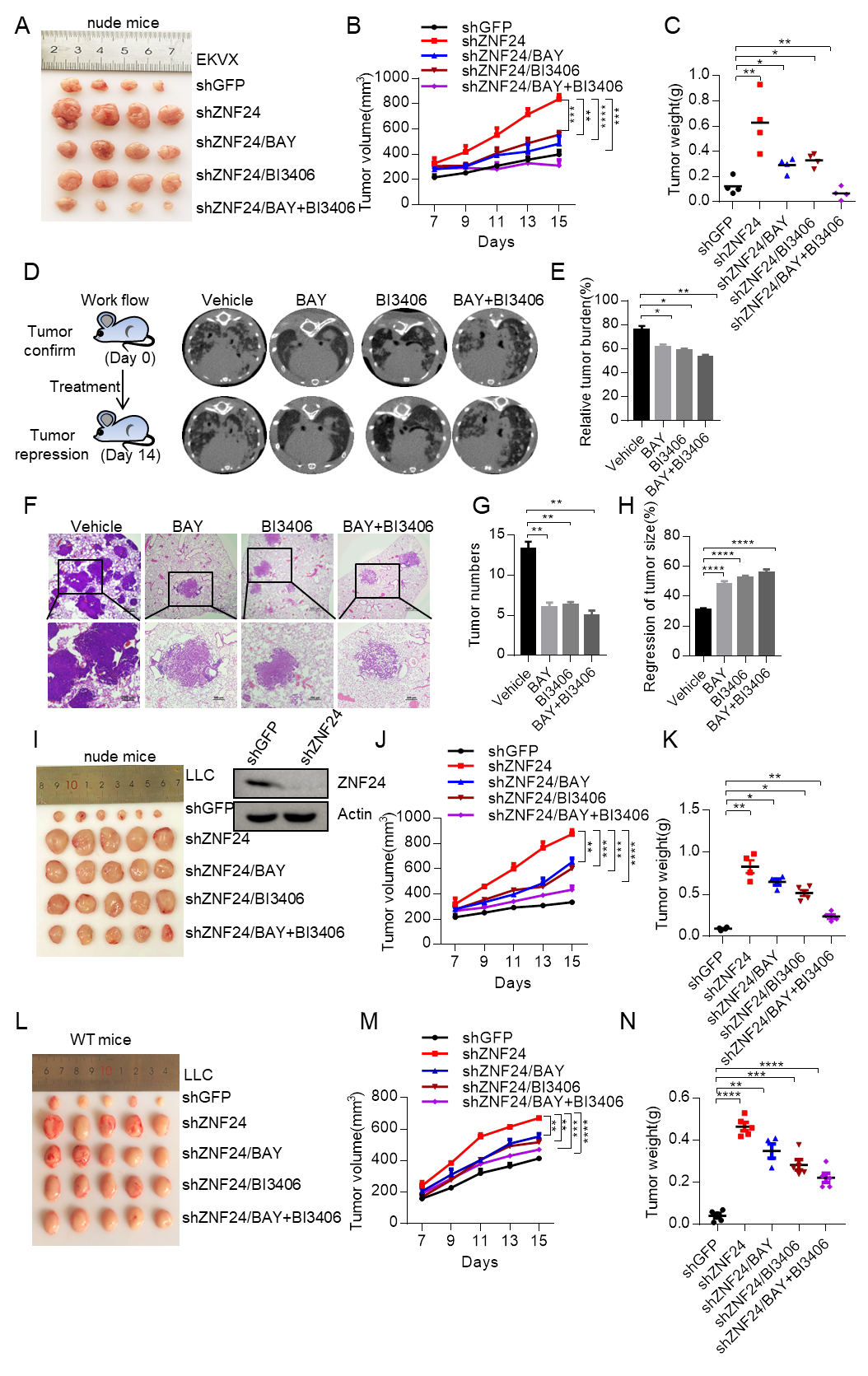
Figure S6. Combinational inhibition of KRAS, NF-κB and PD-1 effectively shrinks Kras^G12D^/ZNF24^-/-^ lung cancers. (A-C)** Impact of the growth of EKVX-shZNF24 cells in nude mice treated BAY11-7082 synergizes with BI3406. EKVX-shZNF24 cells 2×10^6^ were inoculated into nude mice. Mice were treated with BAY11-7082 (20 mg/kg/day, intraperitoneal injection), BI3406 (25 mg/kg/day, gavage), and combination for 2 weeks. The xenografts were dissected to images **(A)** and the tumor growth or weight were monitored **(B-C)**. **(D)** Combination of BAY11-7082 and BI3406 is limited efficacy to shrink lung tumor in K-sgZNF24 mice. The recombinant lenti-virus co-expressed Cre and CRISPR/Cas9 to infect Lsl-Kras^G12D^ through nasal inhalation. Tumor burdens were documented with CT. BAY11-7082 (20 mg/kg/day, intraperitoneal injection), BI3406 (25 mg/kg/day, gavage) were administered. **(E)** Quantification of relative tumor burden of mice of **(D)**. **(F)** Representative images of Hematoxylin and eosin (H&E) staining of the lung tissue obtained from different treatment groups. **(G-H)** Quantification of tumor numbers and repression of tumor size of K-sgZNF24 mice lung cancers. **(I-K)** Impact of the growth of LLC-shZNF24 cells in nude mice treated BAY11-7082 synergizes with BI3406. LLC-shZNF24 cells 2×10^6^ were inoculated into nude mice. Mice were treated with BAY11-7082 (20 mg/kg/day, intraperitoneal injection), BI3406 (25 mg/kg/day, gavage), and combination for 2 weeks. The xenografts were dissected to images **(I)** and the tumor growth or weight were monitored (**J-K)**. **(L-N)** Impact of the growth of LLC-shZNF24 cells in wild-type mice treated BAY11-7082 synergizes with BI3406. LLC-shZNF24 cells 2×10^6^ were inoculated into wild-type mice. Mice were treated with BAY11-7082 (20 mg/kg/day, intraperitoneal injection), BI3406 (25 mg/kg/day, gavage), and combination for 2 weeks. The xenografts were dissected to images **(L)** and the tumor growth or weight were monitored **(M-N)**.


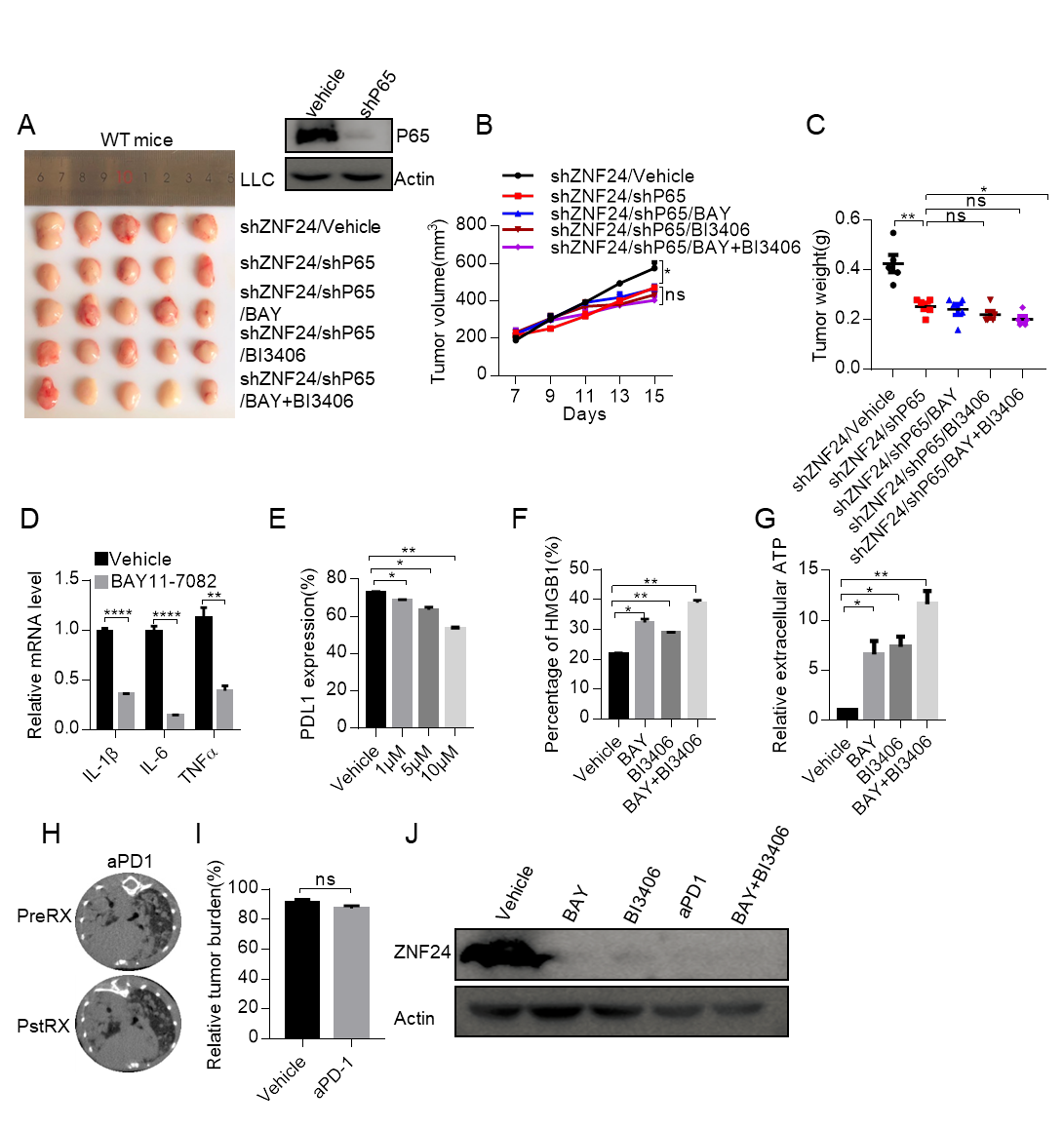
**Figure S7. Combinational inhibition of KRAS, NF-κB and PD-1 effectively shrinks Kras^G12D^/ZNF24^-/-^ lung cancers. (A-C)** Impact of the growth of LLC-shZNF24/shP65 cells in wild-type mice treated BAY11-7082 synergizes with BI3406. LLC-shZNF24/shP65 cells 2×10^6^ were inoculated into wild-type mice. Mice were treated with BAY11-7082 (20 mg/kg/day, intraperitoneal injection), BI3406 (25 mg/kg/day, gavage), and combination for 2 weeks. The xenografts were dissected to images **(A)** and the tumor growth or weight were monitored **(B-C). (D)** RT-qPCR analysis of impact of BAY11-7082 on expression of NF-κB target genes. A549 cells were treated with BAY11-7082 for 24h. *IL-1β*, *IL-6*, *TNFα* expression was quantified through RT-qPCR analysis. **(E)** Impact of PD-L1 expression on BAY11-7082 treatment in A549 cells. A549 cells were treated with BAY11-7082 for 24h. Expression of PD-L1 was detected by flow cytometry. **(F)** Induction of immunogenic cell death by NF-κB inhibitor (BAY11-7082) and/ or KRAS inhibitor (BI3406) in EKVX-shZNF24 cells. EKVX-shZNF24 cells were treated with BAY (2 μM) and/or BI3406 (1 μM) for 24 h. Expression of HMGB1 was determined by flow cytometry. **(G)** Induction of immunogenic cell death by NF-κB inhibitor (BAY11-7082) and/ or KRAS inhibitor (BI3406) in EKVX-shZNF24 cells. Levels of extracellular ATP in treated EKVX-shZNF24 cells measured by luminescence. **(H)** Anti-mouse PD-1 antibody alone is limited efficacy to shrink lung tumor in K-sgZNF24 mice. The recombinant lenti-virus co-expressed Cre and CRISPR/Cas9 to infect Lsl-Kras^G12D^ through nasal inhalation. Tumor burdens were documented with CT. Anti-mouse PD-1 (5 mg/kg, every other day, intraperitoneal) were administered. **(I)** Quantification of relative tumor burden of mice of **(H). (J)** Western blot detecting the knockout efficiency of ZNF24 in K-sgZNF24 mice indicated treated.


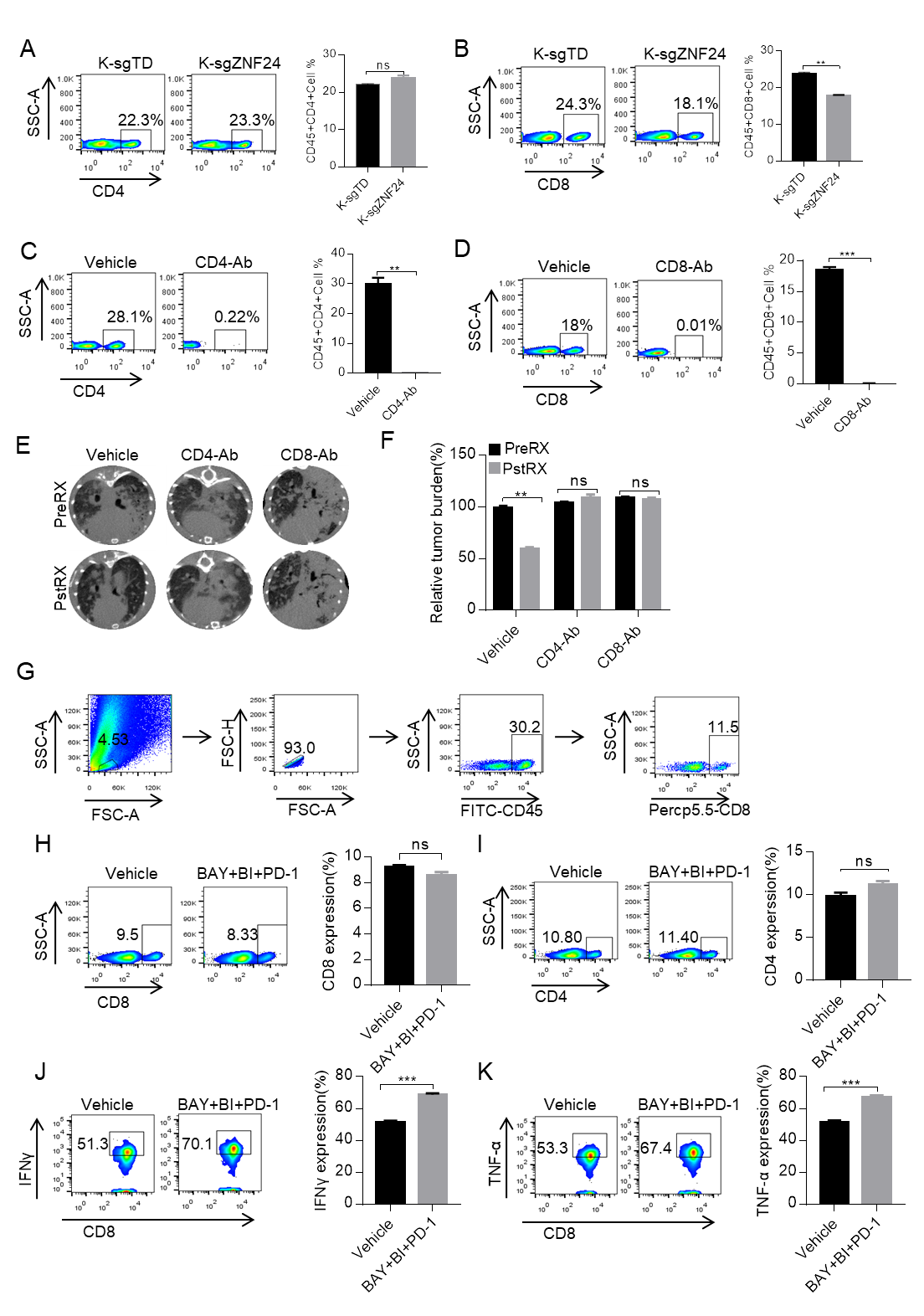
**Figure S8. Combinational inhibition of KRAS, NF-κB and PD-1 effectively shrinks Kras^G12D^/ZNF24^-/-^ lung cancers. (A-B)** Impact of infiltration of CD4+ T cells and CD8+ T cells in K-sgTD and K-sgZNF24 mice. **(C)** Deletion of CD4+ T cells by CD4 antibody in K-sgZNF24. CD4+ T cells in peripheral blood were detected after 2 weeks of treatment. **(D)** Deletion of CD8+ T cells by CD8 antibody in K-sgZNF24. CD8+ T cells in peripheral blood were detected after 2 weeks of treatment. **(E)** Impact of CD4 T cells and CD8 T cells on combinational treatment with BAY11-7082, BI3406 and PD-1 antibody in K-sgZNF24 mice. K-sgZNF24 mice were treated with combination (BAY11-7082, BI3406 and PD-1 antibody) or combinational plus CD4/8 antibody. **(F)** Quantification of tumor burden of mice of Combination of **(E)**. **(G)** Gating strategy for analyzing CD4 and CD8 positive T cells in lung tissues. **(H)** Impact of the combination of BAY11-7082, BI3406, anti-PD-1 (designated BAY+BI+PD-1) on infiltration of CD8+ T cells in lung cancer. Lung tissue were dissected for analyze the infiltration of CD8+ T cells in tumors by flow cytometry. **(I)** Impact of the combination of BAY11-7082, BI3406, anti-PD-1 (designated BAY+BI+PD-1) on infiltration of CD4+ T cells in lung cancer. Lung tissue were dissected for analyze the infiltration of CD4+ T cells in tumors by flow cytometry. **(J-K)** Combinational treatment with BAY11-7082, BI3406 and PD-1 antibody (designated BAY+BI+PD-1) activated expression of effector cytokine in tumor infiltrating CD8+ T cells. Tumor-infiltrating CD8+ T cells were intracellularly stained for FACS analysis of expression of IFNγ and TNF-α. Bars are represented as mean ± SEM of the indicated number (n) of repeats. *P<0.05, **P<0.01, and ***P<0.001 by Student’s t-test.


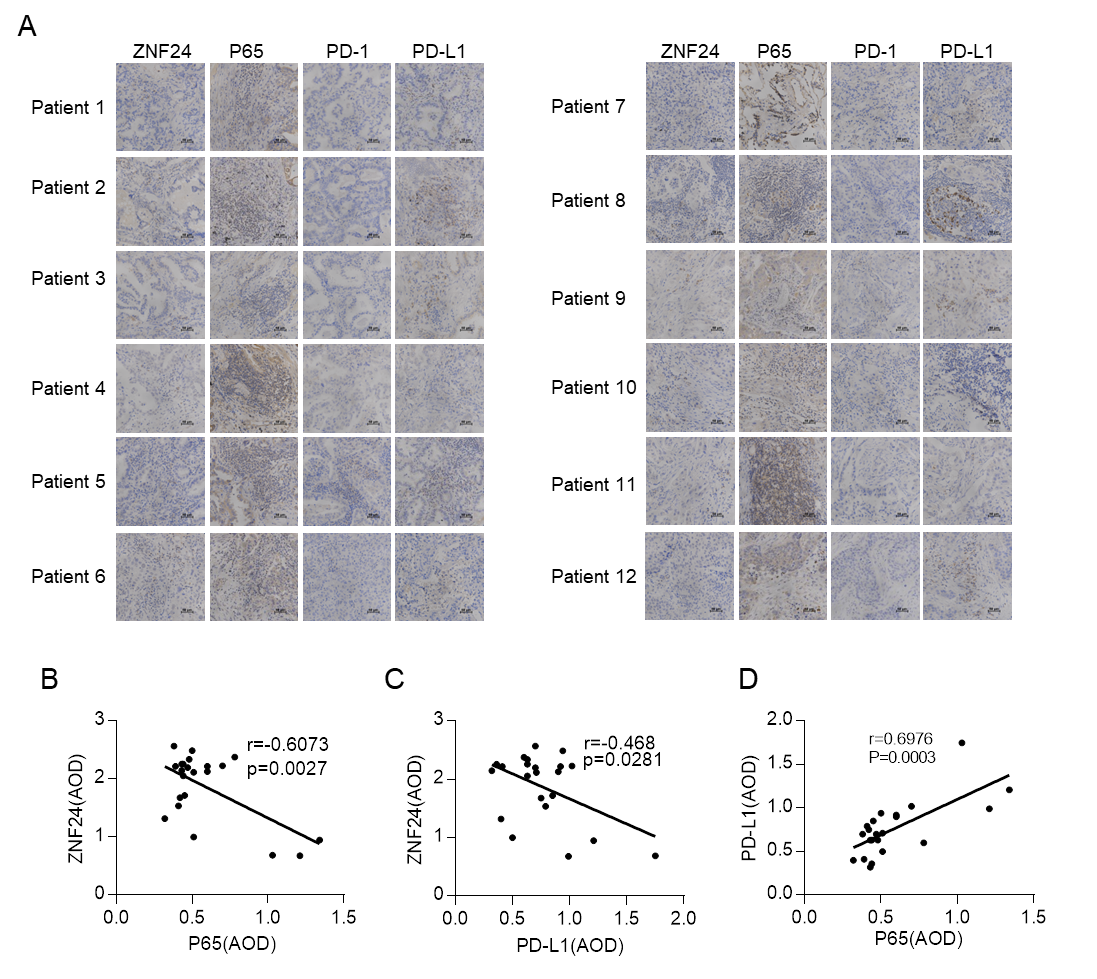
**Figure S9. ZNF24-NF-κB signaling axis is clinically relevant. (A)** Expression of human ZNF24, P65, PD-1, PD-L1 in lung cancer patients revealed through IHC staining. **(B)** Correlation between expression of ZNF24 and P65. Expression data of lung cancer patients from IHC staining by Average Optical Density (AOD) with Image J. **(C)** Correlation between expression of PD-L1 and ZNF24. Expression data of lung cancer patients from IHC staining by Average Optical Density (AOD) with Image J. **(D)** Correlation between expression of PD-L1 and P65. Expression data of lung cancer patients from IHC staining by Average Optical Density (AOD) with Image J.
